# Thermoelectric materials and applications for energy harvesting power generation

**DOI:** 10.1080/14686996.2018.1530938

**Published:** 2018-11-14

**Authors:** Ioannis Petsagkourakis, Klas Tybrandt, Xavier Crispin, Isao Ohkubo, Norifusa Satoh, Takao Mori

**Affiliations:** a Laboratory of Organic Electronics, Linköping University, Norrköping, Sweden; b Center for Functional Sensor & Actuator (CFSN) and International Center for Materials Nanoarchitectonics (WPI-MANA), National Institute for Materials Science (NIMS), Tsukuba, Japan; c Graduate School of Pure and Applied Sciences, University of Tsukuba, Tsukuba, Japan

**Keywords:** Thermoelectric, organic, energy harvesting, thin film, 50 Energy Materials, 210 Thermoelectronics / Thermal transport / insulators

## Abstract

Thermoelectrics, in particular solid-state conversion of heat to electricity, is expected to be a key energy harvesting technology to power ubiquitous sensors and wearable devices in the future. A comprehensive review is given on the principles and advances in the development of thermoelectric materials suitable for energy harvesting power generation, ranging from organic and hybrid organic–inorganic to inorganic materials. Examples of design and applications are also presented.

## Graphical Abstract

**Figure:**
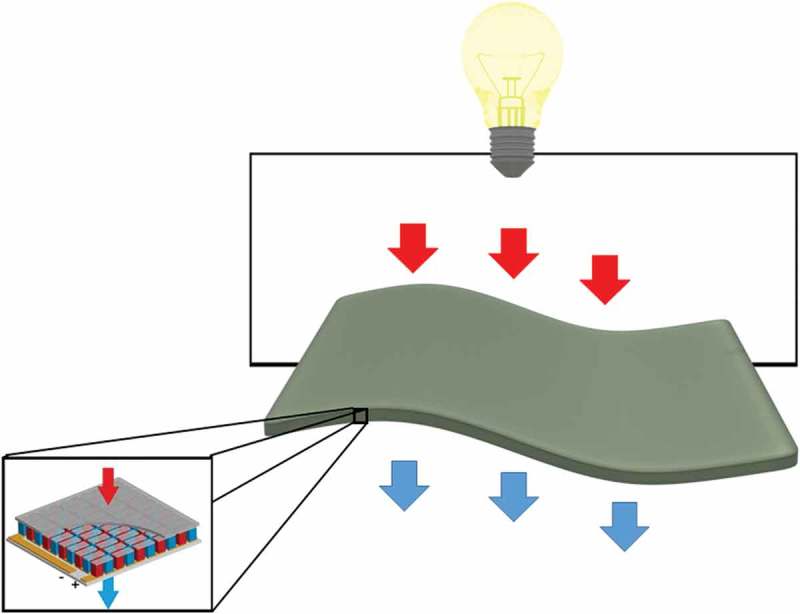

## 1. Introduction

The expected future IoT (internet of things) society may require approximately a trillion sensors for ubiquitous sensor networks, etc. [1]. There is a great need to develop technologies that can power these sensors without the need to replace batteries. Much effort is put on developing energy harvesting technologies that can dynamically harvest various forms of energy from the environment and convert it to electricity. These are technologies such as thermoelectrics, piezoelectrics, magnetoelectrics, which utilize thermal energy, mechanical vibrations, electromagnetic waves, respectively [2].

Thermoelectrics utilize the Seebeck effect for solid state conversion of heat to electricity. A gauge representing the performance of thermoelectric materials is given by the figure of merit, *ZT = S^2^σT/κ*, where *S* is the Seebeck coefficient, σ is electrical conductivity, *κ* is thermal conductivity, and *T* is temperature. The larger *ZT* is, the closer the maximum conversion efficiency approaches the ideal Carnot efficiency. The typical tradeoff between the Seebeck coefficient and electrical conductivity, and somewhat paradoxical requirement of a material conducting electricity but as little thermal conductivity as possible, have hindered the enhancement of *ZT*. Therefore, various novel principles and materials are being actively developed to improve *ZT* and the overall thermoelectric performance [3–5].

The materials and applications near room temperature are especially expected to be useful for energy harvesting [6–8]. One prominent application is to try to use body heat by wearable thermoelectric modules to power mobile devices and sensors. Although the electrical power requirements for such devices and sensors is not so high, for example only 0.1 mW for some electronic tracking tags [9], there are many efforts going on to develop devices which require less power, like spintronic devices for example [10]. However, the effective temperature differences which can be utilized from body heat is small, and therefore, the thermoelectric module should be coupled with a battery, or further enhancement of thermoelectric performance needs to be realized. Since thermoelectric energy harvesting can be continuous, even a 10 μW generator can power up a 100 mW-class IoT device that uses a battery and senses or transmits for one second in every 3 h. In any case, the higher the thermoelectric performance, the wider are power generation applications of thermoelectrics.

Another typical requirement for wearable applications is for the materials/power generation modules to be flexible, and as such there is a great activity ongoing in particular, to develop organic thermoelectric materials for this end.

In this article, we review the fundamentals and development of state-of-the-art organic thermoelectric materials. We also include research efforts for organic–inorganic hybrids and inorganic materials, and also some application designs for utilizing thermoelectric power generation for energy harvesting applications.

## 2. Polymer thermoelectrics

### 2.1. Motivation for polymer thermoelectrics

**Figure 1.**
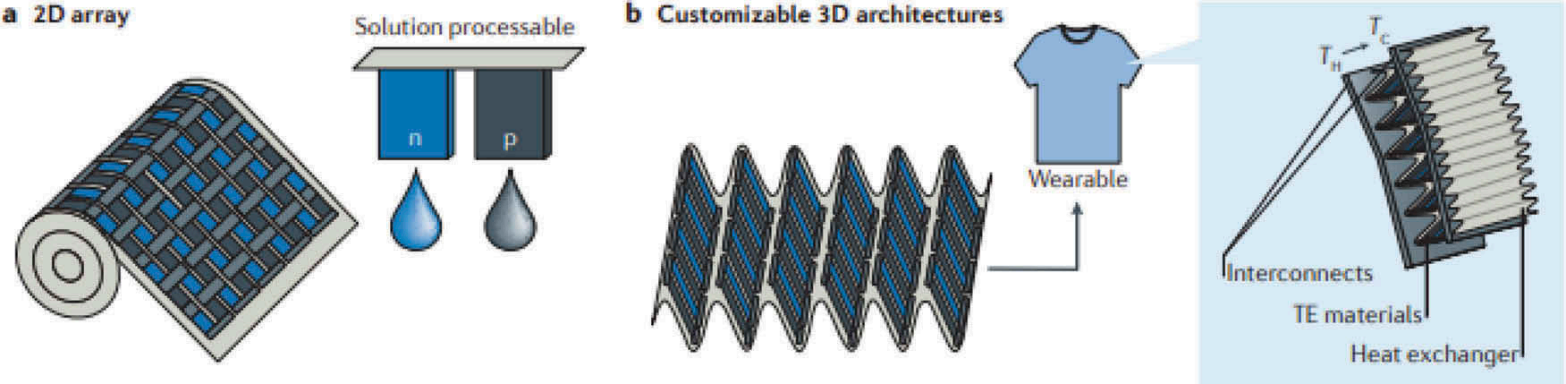
(a) A printed polymer thermoelectric device from a roll-to-roll process from solution processable p-type and n-type polymers. (b) 2D printed arrays can be folded into flexible and lightweight 3D structures ideal for wearable applications. Reprinted by permission from Springer [11], Nature Materials Reviews, Copyright 2016.

Conducting polymers have appeared only recently as candidates for thermoelectric energy harvesting technologies [11]. In comparison to conventional inorganic semiconductors, semiconducting polymers have a relatively lower thermal conductivity (*κ*_pol_ ~ 0.1–1 Wm^−1^K^−1^, *κ*_inorg_ ~ 10–100 Wm^−1^K^−1^), while their electrical conductivity can be as high as *σ *~ 1000 S/cm. This unique combination of properties makes this class of material a potential candidate for thermoelectric applications. Additionally, most polymers have the major advantages of being printable, flexible and moldable, in comparison to their inorganic rigid counterparts. This enables the fabrication of printed organic thermoelectric devices as illustrated in Figure 1. Printing technology is a low-cost manufacturing technique that enables the replication of a large number of legs in order to produce a large thermovoltage. This is achievable in a single manufacturing process without high temperature treatment and on flexible substrates. As a result, polymer thermoelectrics are candidates for self-powered devices in the Internet of Things or in powering everyday appliances (i.e. cellphones). However, the main disadvantage of polymers is their lower thermoelectric efficiency in comparison to their inorganic compartments. Nevertheless, it has been shown [11–13] that with rigorous and strategic material design, polymers can rival the efficiencies of their inorganic brothers. In the next parts of this review, several reported strategies on the enhancement of the thermoelectric properties of polymers are going to be reviewed.

### 2.2. Conducting polymers: basic concepts

**Figure 2.**
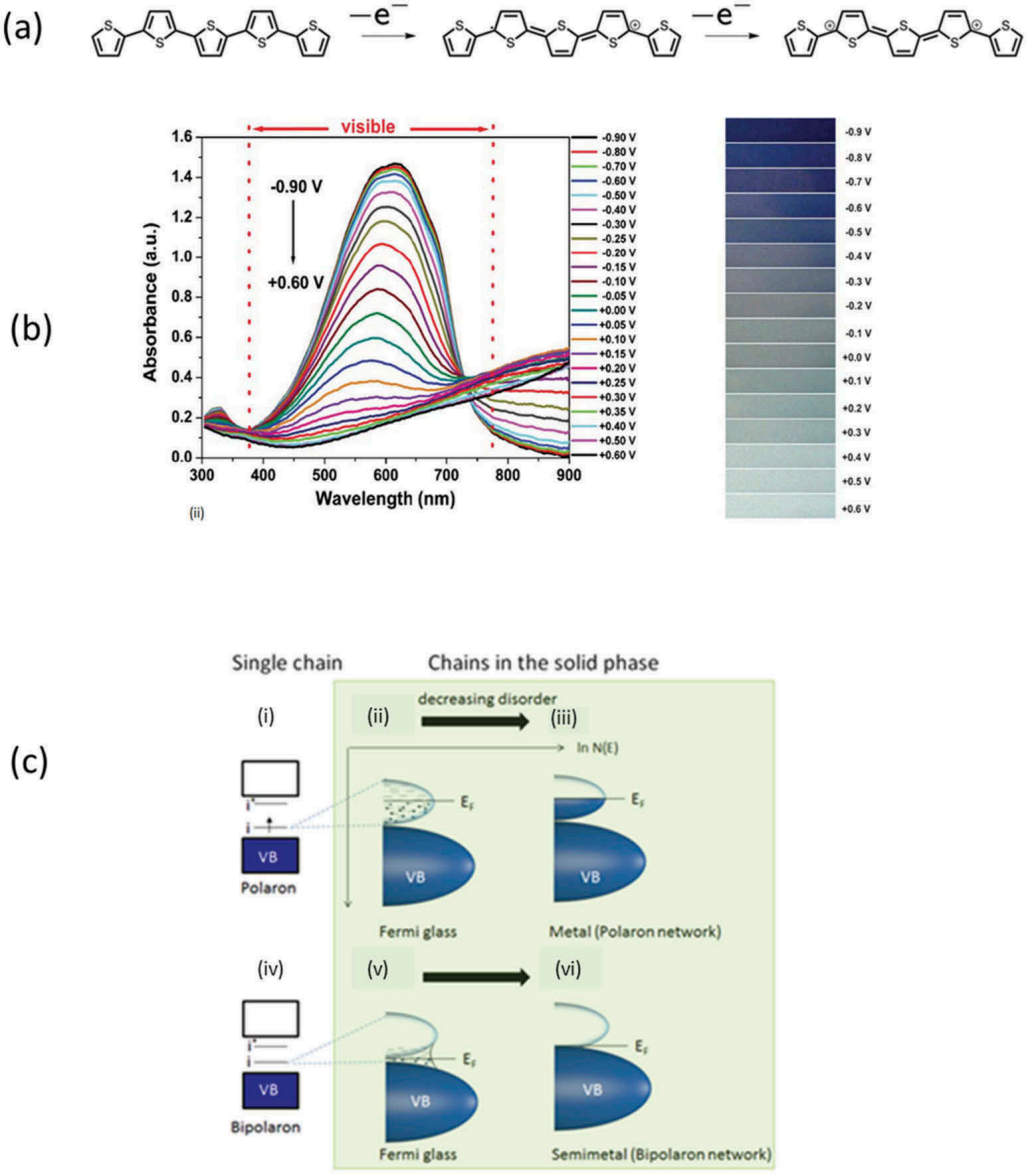
(a) The oxidation of an oligothiophene that forms a polaron (center) and a bipolaron (right). Reproduced from [17] with permission of The Royal Society of Chemistry. (b) UV-Vis spectra (left) and the respective color change (right) in PEDOT, where the doping levels are electrochemically tuned. Reproduced from [18] with permission of The Royal Society of Chemistry. (c) The dependence of the density of states of the conducting polymers to the order of the system. As disorder decreases, the system changes from a fermi glass to a metal (for a network of polarons i.e. Polyaniline) or from a fermi glass to a Semimetal (for a bipolaron network). Reprinted by permission from Springer: Nature Materials, [21], Copyright 2014.

The electronic and electrical properties of conducting polymers originate from their unique chemical structure; they are π-conjugated systems. In such systems, the π-orbital electrons of the polymer backbone atoms are delocalized along the polymer chain, forming extended molecular π-orbitals. This electron delocalization heavily influences the electronic properties of the polymer. Conjugated polymers can either be oxidized by the removal of electrons (p-doping/oxidation process) or reduced by the addition of electrons (n-doping/reduction process). In comparison to inorganic thermoelectrics, where the doping occurs by introducing impurities to the system in a ppm level [14], in (semi)conducting polymers, the charge carrier concentration is usually up to 30% and it is directly linked to the oxidation levels and the chemistry of the material, leading to a completely different and new electronic structure. In the doping process the electronic charges on the polymer backbone are electrostatically stabilized by the inclusion of counter ions of the opposite charge. Upon doping, the chain conformation (bond lengths) around the charge is locally distorted, forming a charged quasiparticle labeled polaron (Figure 2(a)). One distinctive feature of polarons is that they carry a spin. In some polymers, the additional doping of the system leads to the formation of bipolarons, which are more energetically favorable than polarons. At even higher dopant concentration, the number of bipolarons is further increased, resulting in the formation of widened bipolaron bands in the material, that decrease the optical band gap of the system [15,16]. The formation of bipolarons can be probed by spin measurements as bipolarons lack spin. Doping also impacts on the optical properties of conducting polymers. One example is poly(3,4-ethylenedioxythiophene) (PEDOT), which color changes from dark blue in the undoped state to transparent in the heavily doped state (Figure 2(b)). This is reflected in the absorption spectra as a peak at 600 nm for the undoped state, without the presence of any (bi)polaron bands in the infrared region. When the material starts oxidizing, new features appear in the band gap of the materials: first a localized peak around 900 nm which is tentatively attributed to polaron formation, and secondly a broad absorption in the IR. The latter is typical of quasi-free electrons and it indicates that the optical band gap becomes vanishingly small [15].

In conducting polymers, the main charge transport mechanism is thermally activated hopping transport in which the charge carriers ‘hop’ between the different units on an interchain and intrachain level [12,19,20]. These hopping sites are the extended π-systems formed between the chain units, which are inherently conducting. In highly doped conducting polymers, these conductive formations are called ‘metallic’ islands and are dispersed inside the amorphous polymer matrix. Consequently, the electronic structure and electronic properties of doped polymer materials are highly dependent on the structural properties such as the degree of crystallinity and the chain orientation [19,21]. In a material with a high degree of crystallinity, π-orbitals can overlap in between chains, resulting in a much extended π-system. Hence, larger ‘metallic islands’ are formed in the amorphous matrix and charge carriers are more mobile in the material. Also, the long chains benefit from the intrachain hopping transport resulting in an enhanced charge carrier mobility. The interchain transport mechanism could occur either normal to the main axis of the chain and in the direction of the backbone plane or along the π-stacking direction. The first mechanism would imply a long-range hopping to overcome C-H extremities of the molecular backbones. The second mechanism is favorable since the π-orbitals of two adjacent chains are at a closer distance (~3.5 Å), thus leading to an increased hopping rate between the sites. In a conducting polymer material where all three aforementioned structural parameters are being satisfied at the intrachain and interchain levels, the π-orbitals recombine into a much extended π-system, called a ‘bipolaron network’.

The density of states (DOS) of a conducting polymer describes the number of available states which can be occupied as a function of energy. The Fermi level, E_F_, lies among localized states in the middle of the polaron band for a disordered polaronic polymer solid (Figure 2(c), ii); or between the valence band (VB) and the bipolaron band for a disordered bipolaronic polymer solid (Figure 2(c), v). Both solids can be considered as Fermi glasses. E_F_ within localized states implies that the carriers are localized and temperature activated hopping is needed for the transport between localized states. In such situation, σ→0, when T→0. However, in the case of a bipolaron network, where the bipolaron levels are overlapping, the material can behave as a semimetal, as this overlap of the energy levels is shifting the position of the Fermi level (Figure 2(c), vi). In fact, a polaronic network, like polyaniline, can shift from a Fermi glass to a metallic behavior with increasing order of the material. A bipolaron network in PEDOT can shift from a Fermi glass to a semimetal with increasing order in the system [12,21,22].

With respect to the thermoelectric properties, an ideal conformation of the 'bipolaron network' benefits the Seebeck coefficient of the material. The Mott’s equation on thermoelectricity dictates that the Seebeck coefficient of a material is analogous to the slope of the (DOS) at the Fermi level, *S *~${\left(\frac{\partial lnDOS\left(E\right)}{\partial E}\right)}_{E_{F}}$. In a doped conducting polymer with a ‘polaron network’, the slope of the DOS at *E*_F_ is close to zero and the Seebeck coefficient is expected to be small. On the contrary, for a ‘bipolaron network’, the slope of the DOS at *E*_F_ is steeper and the Seebeck coefficient of the material is higher than for a polaron network. Also the crystallinity or degrees of order will have a significant impact on the DOS. With energy disorder, the DOS broadens and softens at the band edge; as a consequence, the slope of the DOS at *E*_F_ decreases and the Seebeck coefficient for disordered material is low. This is why the Seebeck coefficient in conducting polymers (typically amorphous or para-crystalline) is lower than crystalline inorganic semiconductors. Consequently, proper and detailed material design is needed in order to achieve high performance thermoelectric materials [12,21,22].

### 2.3. Optimization strategies for p-type thermoelectric polymers

#### 2.3.1. Tuning of the doping level

In their birth, conducting polymers were not envisioned as prospective thermoelectric materials. The very first Seebeck measurements on semiconducting polymers were conducted on polyacetylene to understand the nature of the charge carriers. Park et al. varied the doping level of polyacetylene, showing that the Seebeck coefficient decreases for high carrier concentrations [23]. However, the first actual demonstration of the potential for polymers in thermoelectrics was given by Hiroshige et al. [24], who reported PPV doped with iodine with a figure of merit ~ 0.1. Xuan et al. [25] optimized the power factor of the semiconducting poly(3-hexylthiophene) (P3HT) by oxidation with NOPF_6_. The thermoelectric efficiency was clearly limited by the modest electrical conductivity (< 10 S/cm).

**Figure 3.**
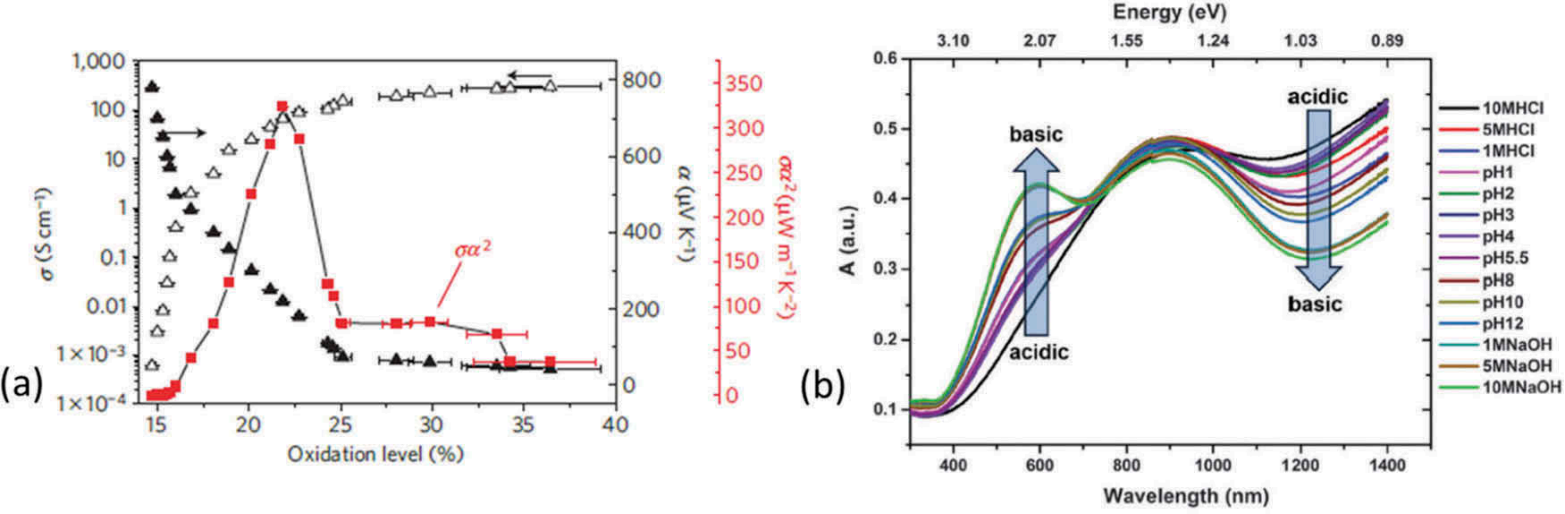
(a) The electrical conductivity, *σ*, the Seebeck coefficient, *α*, and the power factor, *σα^2^*, versus the oxidation levels of the PEDOT:Tos films. Reprinted by permission from Springer: Nature Materials [26], Copyright 2011. (b) The UV-Vis spectra of acid and base treated PEDOT:Tos with respect the pH of the treating solution [29]. Published by The Royal Society of Chemistry.

Later, Bubnova et al. [26] optimized the power factor of the high conductivity polymer, poly(3,4-ethylenedioxythiophene):*p*-toluenesulfonate (PEDOT:Tos) (300 S/cm) upon exposure to reductive vapors. By de-doping the material, the Seebeck coefficient increased while the electrical conductivity decreased, showing a similar behavior as inorganic materials (Figure 3(a)). However, in comparison to inorganic thermoelectric materials, the PEDOT:Tos films exhibited a relatively low thermal conductivity of 0.2–0.25 W/m∙K. The power factor was optimized from 38 to 324 μW/m∙K^2^, which gave a figure of merit of 0.25 at room temperature [26]. This was the second study recognizing the vision of ‘polymer thermoelectrics’.

The degree of doping or dedoping in a material is heavily affected by the strength (electrochemical potential) and the chemical nature of the oxidizing/reducing agent itself as showed by Massonnet et al. [27] upon dipping films of poly(3,4-ethylenedioxythiophene):poly(4-styrenesulfonate) (PEDOT:PSS) in different reducing agents like tetrakis(dimethylamino)ethylene (TDAE) or hydrazine. Wang et al. [28] reported on the optimization of PEDOT films by dipping in the reducing agent NaBH_4_ and enhanced the figure of merit from 0.02 to 0.155 at 385 K. Beside exposing to reducing agents, Khan et al. [29] demonstrated the impact of the pH on the thermoelectric properties of PEDOT films. It was reported that the Seebeck coefficient and the electrical conductivity showed an antagonistic behavior with the pH of the solutions, allowing for optimization of the system. It is believed that the protons can transfer their charge in the π-conjugated system when they covalently bond to the alpha carbon of the ends of the PEDOT chains. The tuning of the oxidation level was further illustrated with absorption spectra (Figure 3(b)), where the acid treated films had a strong bipolaronic wide-arm in the near IR, while the base treated films exhibited a peak at 600 nm typical of neutral PEDOT segment.

Fan et al. [30] followed a sequential washing treatment. The pristine PEDOT:PSS films were initially washed with sulfuric acid, as a means to increase the electrical conductivity of the system, both by oxidation and by changing morphology and composition of the material. Afterwards, the film was exposed to a reducing agent. As a result, the power factor of PEDOT:PSS was enhanced from 0.0045μW/m∙K^2^ to 334 μW/m∙K^2^. Later, in the same year, Jung et al. [31] reported a study on doping engineering of a diketopyrrolopyrrole-based polymer, namely, by unconventional doping through spin coating. The authors reported a power factor of 276 μW/m∙K^2^ for their iron chloride doped polymer, emphasizing that a low dopant volume was crucial for a higher Seebeck coefficient in this polymer.

**Figure 4.**
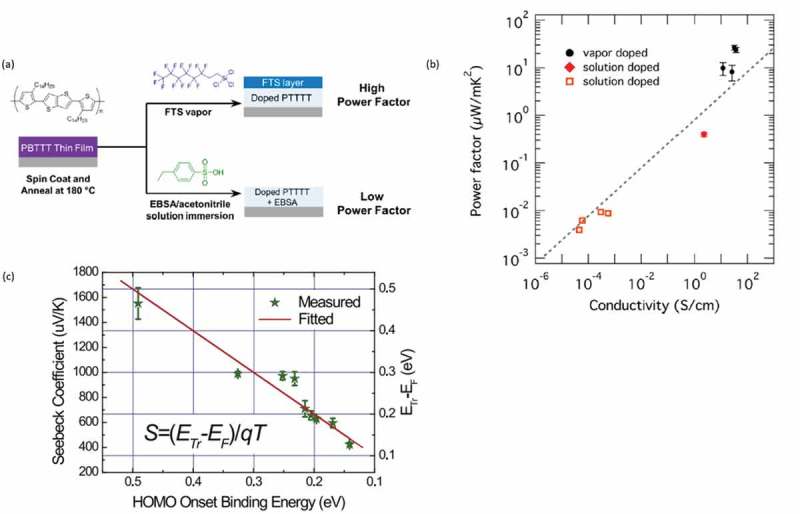
(a) The vapor and solution routes for the treatment of PBTTT. Reprinted (adapted) with permission from [32]. Copyright (2016) American Chemical Society. (b) The power factor vs conductivity for P3HT treated with vapors and solutions. Reprinted (adapted) with permission from [33]. Copyright (2018) American Chemical Society. (c) The Seebeck coefficient vs the HOMO Onset Binding Energy for the F_4_-TCNQ treated P3HT. The linear fit corresponds to the mobility edge model. Reprinted from [34], Copyright (2018), with permission from Elsevier.

The importance of the processing conditions was firmly highlighted by Patel et al. [32], where the authors compared the thermoelectric properties of a semiconducting polymer, PBTTT, as it was doped by either vapor or by immersing in an oxidative solution. They reported that upon exposure of the material to vapors of (tridecafluoro-1,1,2,2-tetrahydrooctyl)trichlorosilane (FTS), the material had a two orders of magnitude higher power factor (100 μW/m∙K^2^) than when it was immersed in a solution of 4-ethylbenzenesulfonic acid (EBSA) (Figure 4(a)). The difference in performance originated from the Seebeck coefficients, with 14 μV/K for the EBSA and 33 μV/K for the FTS treated samples. The authors concluded that this difference in the Seebeck coefficient originated from either an increase in the entropic vibrational component of *S* or from changes in the scattering of carriers in disordered regions in the film. The importance of the doping processing conditions was also underlined in the work of Lim et al. [33]. for P3HT doped by F_4_TCNQ (small molecule). It was reported that systems doped with vapor of the small molecule had a higher power factor than those doped through dipping in a solution (Figure 4(b)). This discrepancy was attributed to the high electrical conductivity for the vapor-treated material. Meanwhile, Zou et al. [34] reported for the same system (P3HT/F_4_TCNQ) a linear dependence between the Seebeck coefficient and the band edge of P3HT thin films (Figure 4(c)).

**Figure 5.**
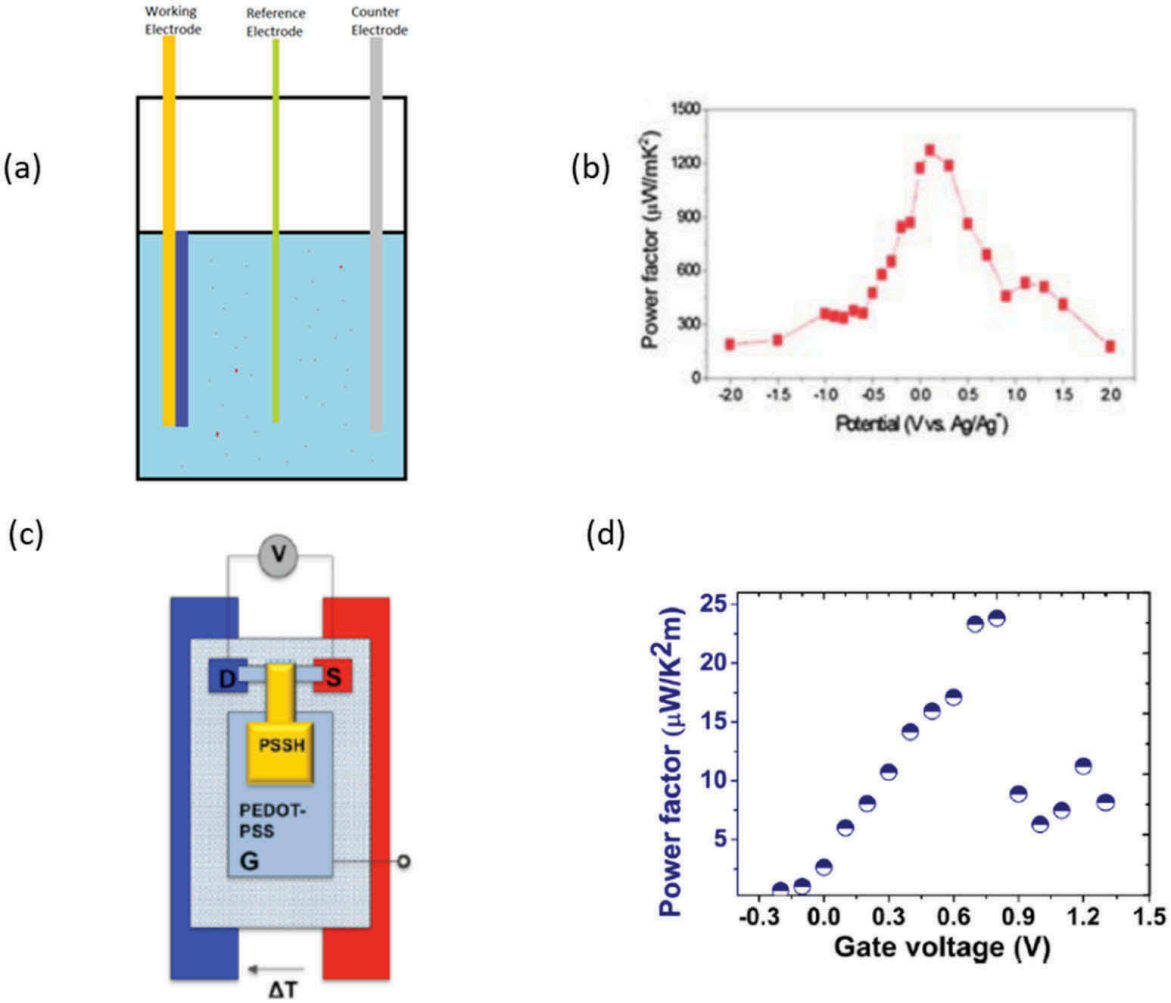
(a) A conventional electrochemical setup; with yellow is the working electrode that has the PEDOT (blue) deposited on top, with green is the reference electrode (usually Ag/AgCl) and with gray is the counter electrode. (b) The power factor of PEDOT:Tos films plotted against the applied voltage from chronocoulometry. Reproduced from [35] with permission of The Royal Society of Chemistry. (c) Organic electrochemical transistor used for the optimization of the thermoelectric properties of PEDOT:PSS and the resulting power factor (d) plotted versus the applied gate voltage. Reprinted with permission from [36]. Copyright 2012 American Chemical Society.

All the aforementioned works involve a tuning of the doping levels with a conventional chemical redox reaction. An alternative approach for doping optimization is through electrochemical methods, which offer a better control for fine-tuning the doping levels by changing the electrochemical potential of the polymer and measuring a current corresponding to the amount of doping charges. A typical electrochemical setup is presented in Figure 5(a). Park et al. [35] optimized the doping levels of PEDOT:Tos to a record power factor of 1270 μW/m∙K^2^ (Figure 5(b)). Another use of electrochemical optimization was reported by Bubnova et al. [36] where an organic electrochemical transistor (Figure 5(c)) was used to optimize the thermoelectric properties of PEDOT:PSS. In such a device, the power factor was optimized by applying a gate voltage in the configuration (Figure 5(d)). Electrochemistry can also be used to directly polymerize conducting polymers through electropolymerization. Parameters such as temperature, current density, frequency, electrolyte, counterions (e.g. Zhang et al. [37] uses sulfated poly(β-hydroxyethers) counterions for PEDOT) can be used to modify the morphology and film properties. Culebras et al. [38] published a report on electropolymerized PEDOT with conductivity of ~ 1000 S/cm and an optimized figure of merit of 0.22 at room temperature.

#### 2.3.2. Tuning of the macromolecular structure

The electronic properties of conducting polymers are heavily based on their macromolecular design [39]. Parameters like the monomer unit structure or the molecular weight of the polymer can strongly alter the electrical behavior of the material, as they affect the electronic structure and therefore the charge transport and the thermoelectric properties. One of the first attempts to systematically investigate the effect of the macromolecular design on the thermoelectric properties was reported from Levesque et al. [40] in the 2000’s. The authors investigated the effect of the chemical structure of polycarbazoles on the thermoelectric properties. Their most efficient material was a copolymer of thiophene with carbazole with electrical conductivities in the order of 10^−2^ S/cm, but relatively high Seebeck coefficient around 400–500 μV/K. The influence of the macromolecular structure was further underlined in the later studies of Li et al. [41] and Wang et al. [42]. In the first study, the authors synthesized derivative copolymers of poly(bisdodecylquaterthiophene) (PQT12) with thiophene and EDOT rings and inserted a sulfur atom between the thiophene rings and the dodecyl chains. They reported that the films of the derivative with the thiophene and the sulfur atom exhibited the highest power factor (~ 10 μW/m∙K^2^). Meanwhile, Wang et al. synthesized donor-acceptor copolymers and concluded that polymers containing the thiophene units exhibited the highest Seebeck coefficient (334 μV/K) and the highest power factor (13 μW/m∙K^2^).

Another aspect of the macromolecular design that affects the electronic properties of the system is the polymer molecular weight [43]. Fan et al. [44] compared two grades of commercial PEDOT:PSS with different molecular weight of the PEDOT. The authors reported that the PEDOT:PSS grade with the highest molecular weight (PH1000) exhibited both highest Seebeck coefficient and highest electrical conductivity. Longer PEDOT chains result in longer PEDOT ordered domains that facilitate the charge transport and the thermodiffusion of the charge carriers [22,44]. Long chains act as conductive bridges between the crystallites of PEDOT in the thin film, resulting in higher thermoelectric performance but also in a semimetallic behavior of the film [22].

#### 2.3.3. Tuning of the morphology

The polymer morphology in thin films heavily influences the electronic properties of the material [45]. In principle, conducting polymers with higher degree of crystallinity are more electrically conducting, as the charge transport between the conducting, crystalline, metallic islands is facilitated [19,46]. However, this also benefits the Seebeck coefficient of the system, as the charge carriers can diffuse easier under a thermal gradient, resulting in a high Seebeck coefficient [21,22]. Therefore, morphology can also act as another lever to tune the thermoelectric efficiency of polymers. The very first investigation in this aspect came from Park et al. on their studies on polyacetylene, where they found that oriented heavily doped polyacetylene has a higher thermopower than non-oriented polyacetylene. Particularly, polyacetylene exhibited a low Seebeck coefficient (< 20 μV/K) that linearly depended on the temperature, inherent to a typical metallic behavior [47].

**Figure 6.**
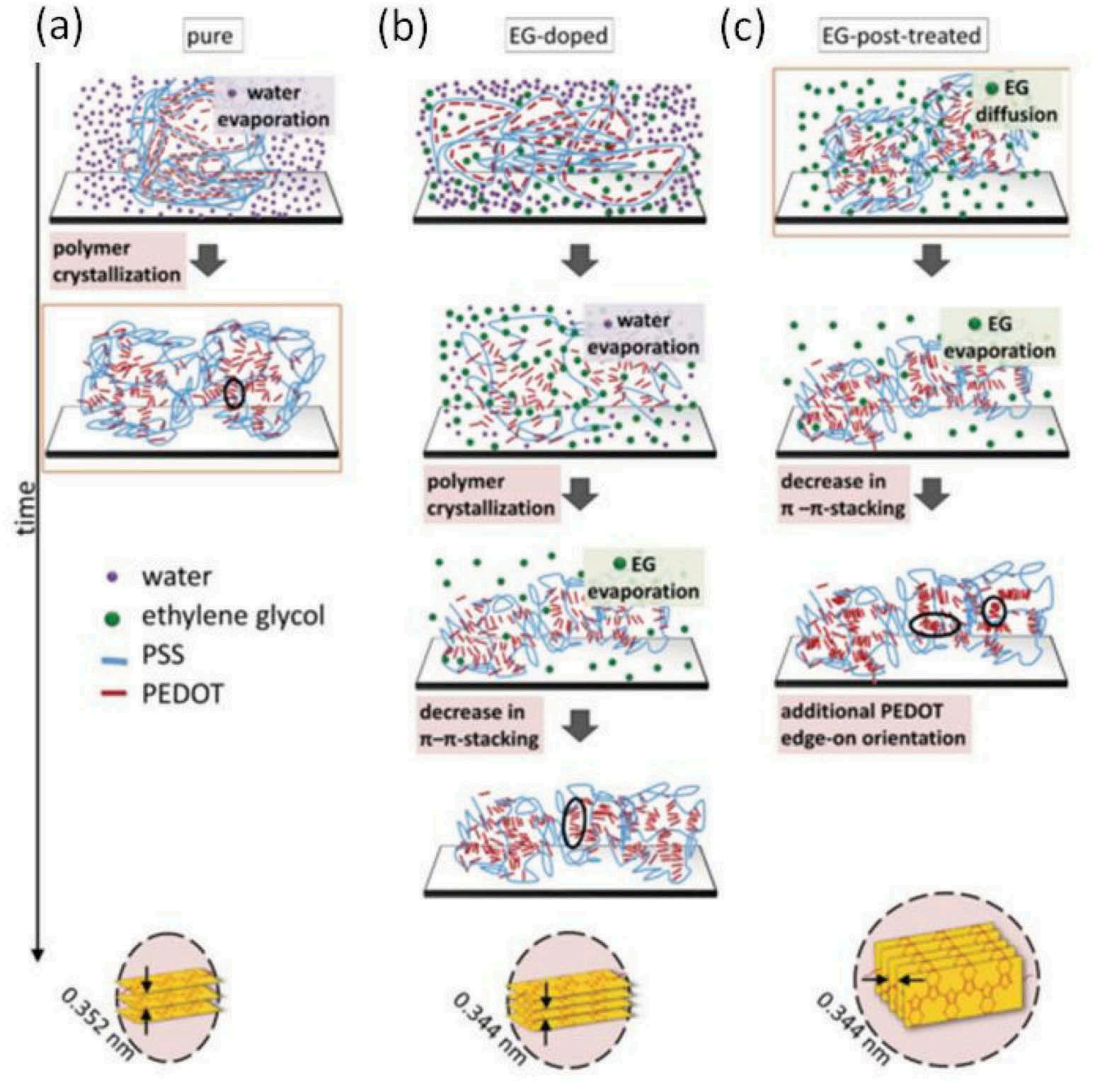
The effect of plasticizing agents like EG on the PEDOT:PSS structure. When EG is added to the PEDOT:PSS material (b), the crystallization process is slowed down resulting in better stacked chains, in comparison to the pure (a) material. When the thin films are further treated with an EG bath, the system recrystallizes and shift from an face-on to an edge-on configuration. Reprinted by permission from Wiley [51], Copyright 2015.

In more recent years, Kim et al. [48] have measured the Seebeck coefficient of the PEDOT:PSS and its behavior versus temperature. It was reminiscent of that of a heavily doped semiconductor in the metallic regime of the Mott’s transition. In comparison with other conducting polymers, PEDOT:PSS is solution processible and can be printed in films showing a high electrical conductivity (> 1000 S/cm). Jiang et al. [49] pinpointed that the addition of a high boiling point solvent improved the figure of merit of the conducting polymer due to an enhanced electrical conductivity. The effect of the high boiling point solvents such as dimethylsulfoxide (DMSO) and ethylene glycol (EG) on the thermoelectric properties of PEDOT:PSS was especially highlighted in the work by Kim et al. [50] After bathing the PEDOT:PSS films in DMSO and EG to remove any excess of insulating PSS, they managed to enhance the polymer power factor to 469 μWm^−1^K^−2^. Combined with a relatively low thermal conductivity of 0.42 Wm^−1^K^−1^, the figure of merit reached 0.42. Palumbiny et al. [51] later demonstrated that this kind of treatment enhances the thin film crystallinity, as the high boiling point solvents act as plasticizers and slow down the crystallization kinetics. As a result, the polymer chains are allowed to rearrange in the thin film, increasing the degree of crystallinity and facilitating the charge transport in the system (Figure 6) [51–53].

Nevertheless, the origin of these interesting thermoelectric properties was still unclear. A first explanation was provided by Bubnova et al. [21] proposing that PEDOT derivatives with increased crystallinity can behave as semimetals, thus exhibiting both a high electrical conductivity and a high Seebeck coefficient. In PEDOT systems of high crystallinity, the π-orbitals of the PEDOT units recombine into a bipolaron network, which broadens all the π-orbitals of the polymer, thus increasing the slope at the density of states and the Seebeck coefficient. As a result, vapor phase polymerized PEDOT:Tos films exhibit power factors as high as 454 μW/m∙K^2^. In recent years, more studies support the beneficial effect of crystallinity on the thermoelectric properties of PEDOT derivatives [21,54–57].

**Figure 7.**
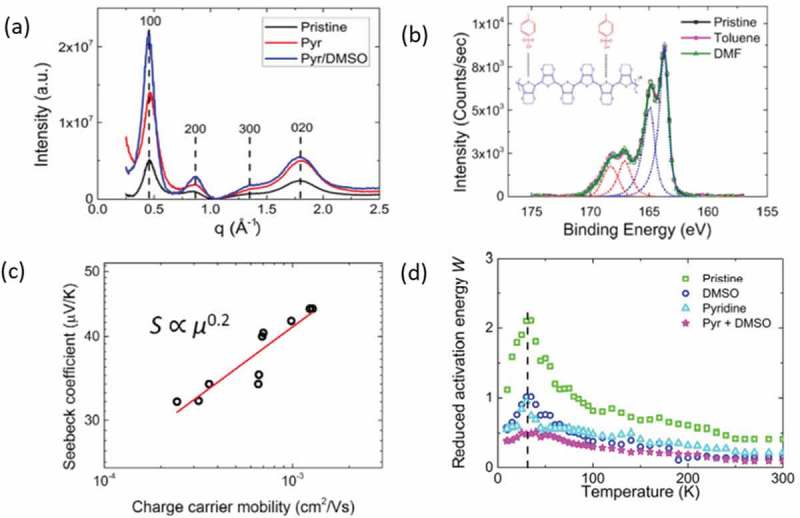
(a) The radially averaged intensity versus the scattering vector q for the various PEDOT:Tos samples. A higher intensity in the peak corresponding to the (100) reflection is respective to a system of higher degree of crystallinity. (b) the XPS spectra of PEDOT:Tos samples with various solvent additives, (c) the extracted relationship between Seebeck coefficient and charge carrier mobility with constant oxidation levels and (d) the Reduced activation energy W versus Temperature, where the Mott transitions are observed. Crystallinity and charge carrier mobility are increasing from the Pristine to the Pyr+ DMSO samples. A negative slope of W is represents a semiconducting material, while constant W is respective of a semimetal [22,46]. Reprinted from Organic Electronics [22]. Copyright 2018, with permission from Elsevier.

The relationship between charge carrier mobility, thin film crystallinity, electrical and thermoelectric properties of conducting polymers was further elucidated by the works of Petsagkourakis et al. [22,46] on PEDOT:Tos thin films. High boiling point solvent additives (DMSO) and an organic base, pyridine, were used as means to enhance the degree of crystallinity of the PEDOT:Tos thin films (Figure 7(a)). Solvent treatment left the oxidation level of the polymers unaffected, as proven by X-ray photoelectron spectroscopy (XPS) (Figure 7(b)). Hence for the same oxidation level, the conductivity of the samples is directly related to the mobility. A relationship between Seebeck coefficient and charge carrier mobility was extracted for those films, *S *~ *μ*^0.2^ (Figure 7(c)). This behavior was attributed to the enhanced degree of crystallinity, which resulted in delocalization of the charge carriers and extended the band edge at the Fermi level. Additionally, a transition between the semiconducting to the semimetallic behavior was observed as the degree of crystallinity and charge carrier mobility increased (Figure 7(d)).

**Figure 8.**
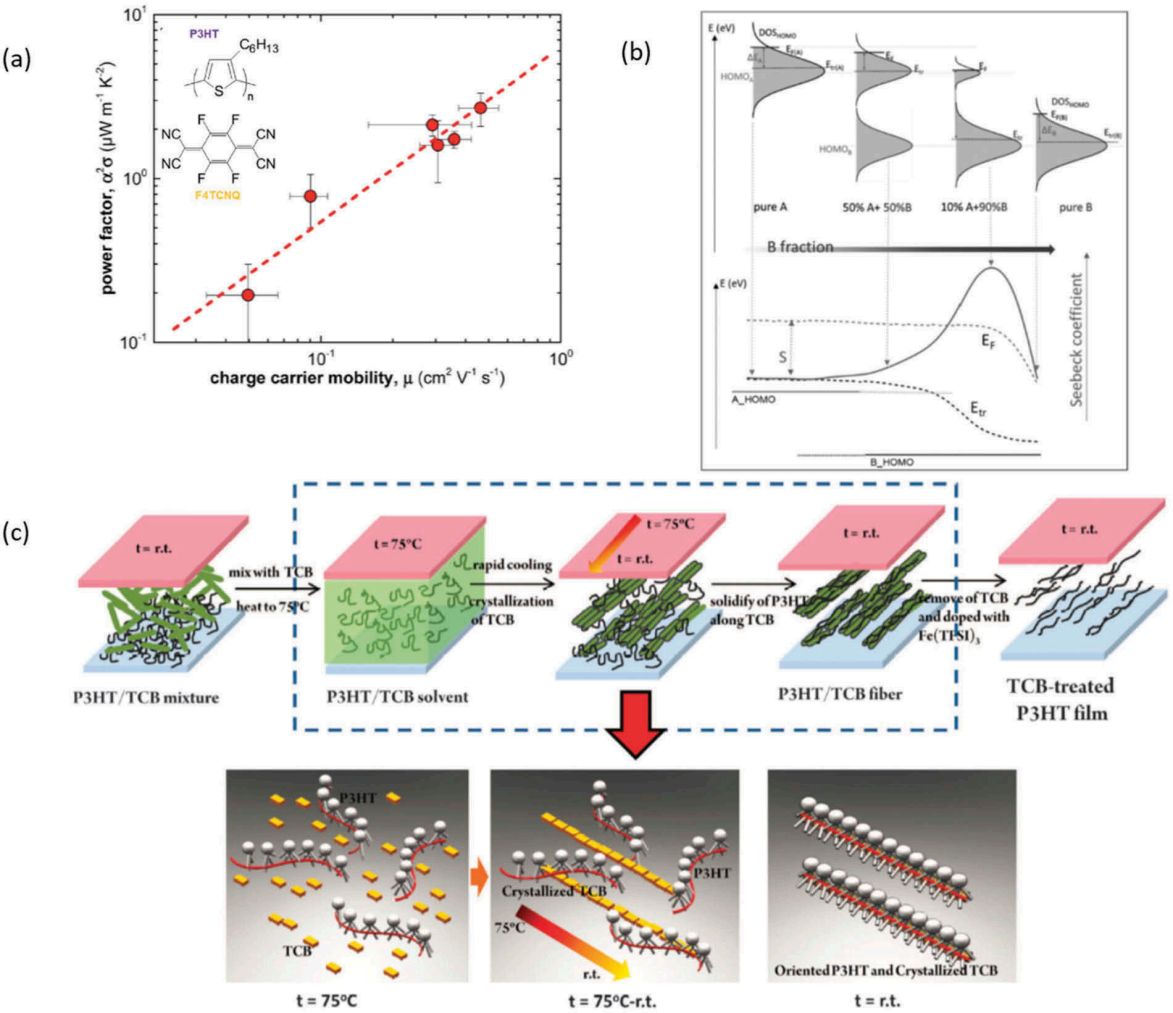
(a) The vapor treatment reported by Hynynen et al. [59] (left) and the extracted power factor/charge carrier mobility (right) for their systems [59]. Published by The Royal Society of Chemistry. (b) The modification of the density of states with the fraction of the polymers in the mixture. Reprinted by permission from Wiley [62], Copyright 2018. (c) The process reported by Qu et al. [60] in using TCB in order to enhance the ordering and thermoelectric properties of P3HT.

The importance of morphology as a lever to control the thermoelectric performance of a material was also underlined from other recent studies on (semi)conducting polymers [58]. Hynynen et al. [59] highlighted that films of P3HT with high degree of crystallinity had a slightly higher Seebeck coefficient and a higher electrical conductivity than disorder films, thus leading to a high power factor. However, the authors also focused on the relationship between power factor and charge carrier mobility of the films. As seen in Figure 8(a), the power factor is proportional to the charge carrier mobility of P3HT, which is directly linked to a gradual increase of the degree of crystallinity. In a similar aspect, Qu et al. [60], used TCB molecules as a template for the structure of P3HT. By using this approach, the authors managed to tune not only the crystallinity of the thin films, but also the orientation of the P3HT chains on the substrate. When the chains were oriented parallel to the substrate (Figure 8(c)), the films exhibited higher electrical conductivity while the Seebeck coefficient was not affected much from the orientation. As a result, a power factor of 40 μW/m∙K^2^ was reported for the films with chains in the parallel orientation. The morphology and properties of polymers can also be tuned by introducing them in mixtures [61]. In that aspect, Zuo et al. [62], mixed P3HT with PTB7, which has a deeper HOMO level. Although the electrical conductivity was decreasing with the % of PTB7, the Seebeck coefficient was increasing, which was attributed to a tuning of the density of states of the mixture (Figure 8(b)).

Mixtures of PEDOT:Tos with PEDOT nanowires were also fabricated by Zhang et al. [63,64]. With this approach, the authors managed to achieve a high figure of merit of 0.44, which they attributed to the formation of a better percolation path formed by the PEDOT nanowires.

### 2.4. Optimization strategies for n-type thermoelectric polymers

**Figure 9.**
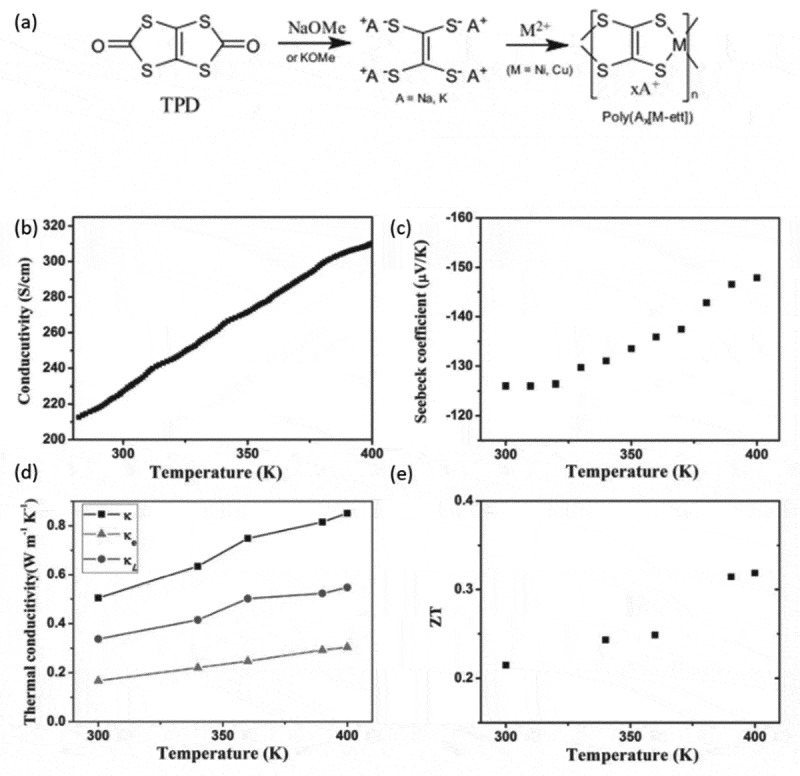
(a) The synthetic route for the metal coordination polymers that Sun et al. reported. Reprinted by permission from Wiley [65], Copyright 2012. (b-e) the electrical conductivity, Seebeck coefficient, thermal conductivity and figure of merit plotted against the temperature for the electropolymerized poly(Ni-ett). Reprinted by permission from Wiley [67]. Copyright 2016.

For an efficient thermoelectric generator both p- and n- type materials are required. Therefore, to realize the dream of an efficient, all-organic thermoelectric generator, n-type polymers are necessary. A major challenge is that n-type polymers are usually unstable since they are oxidized in the atmosphere. Also, most reported systems lack in electrical conductivity in comparison to their p-type equivalents. Consequently, doped fullerene or TTF:TCNQ salts were used as n-type organic thermoelectrics instead of polymers until the 2010’s [12]. The first step towards efficient and stable n-type polymers were reported by Sun et al. [65] in 2012 with the synthesis of metal coordination polymers such as poly(Ni-1,1,2,2-ethenetetrathiolate) depicted in Figure 9(a).

In the metal coordination polymers, a metal is linked to organic conjugated ligands inside the polymer repetitive unit. Interestingly, by changing the transition metal atom of the monomer unit, the sign of the Seebeck coefficient can be changed [65,66]. Concerning the n-type compartment, the poly(Ni-1,1,2,2-ethenetetrathiolate) (poly(Ni-ett)) with potassium counterions exhibited a figure of merit of 0.1 at room temperature. The synthesis of such systems usually results in insoluble dust, thus pellets are fabricated in order to properly characterize the material, which limits the use of such material in flexible thin film electronics [65]. Sun et al. [67], provided a solution to that issue by preparing the poly(Ni-ett) electrochemically. By applying a voltage to a working electrode immersed in a precursor solution, the authors managed to deposit polycrystalline, flexible poly(Ni-ett) films (Figure 9), with ZT ~ 0.3, the highest reported so far for n-type polymer thermoelectrics. The same technique was also used to fabricate one-leg thermoelectric generators of poly(Ni-ett) that exhibited an output power of ~ 0.5 μW at a difference of 12 K [68].

**Figure 10.**
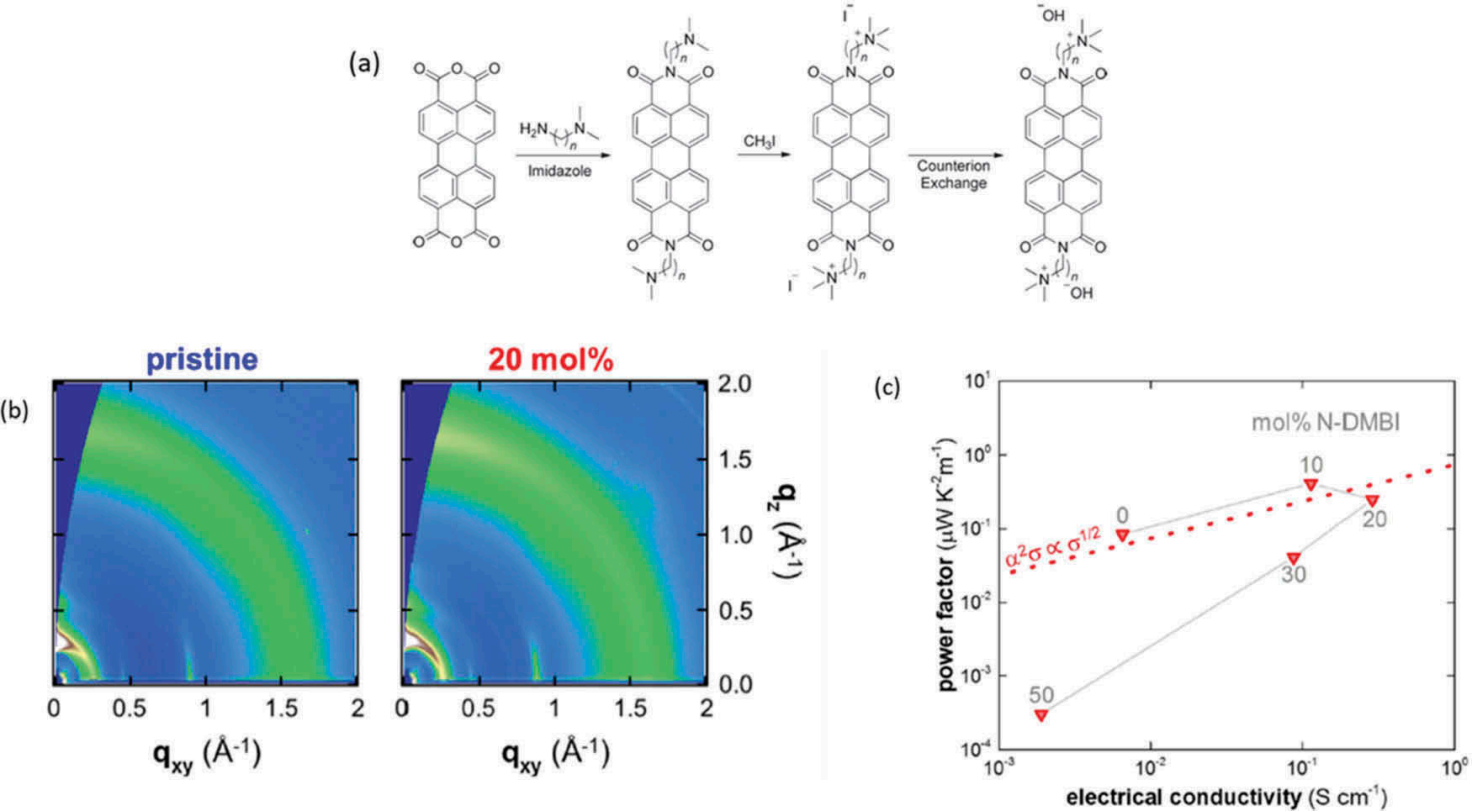
(a) The naphthalene derivatives reported by Russ et al. Reprinted by permission from Wiley [70] Copyright 2014. (b) The GIWAXS patterns for the pristine and doped napthalenediimide derivatives. A higher degree of crystallinity is observed for the doped ones. (c) the power factor vs the electrical conductivity for those systems. Reprinted (adapted) with permission from [73]. Copyright 2018 American Chemical Society.

Soluble n-type all-organic polymers were reported by Schlitz et al. [69] and Russ et al. in 2014 [70]. These naphthalene derivatives (Figure 10(a)) exhibited a power factor of 1.4 μW/m∙K^2^, with an intrinsically low electrical conductivity (0.5 S/cm) but relatively high thermopower (−200 μV/K). Shi et al. [71] reported in 2015 on soluble n-type benzodifurandione paraphenylenevinylidene (BDPPV) derivatives with electrical conductivities as high as 14 S/cm and a power factor of 28 μW/m∙K^2.^ The importance of the backbone structure on the electrical and thermoelectric properties was highlighted, as the introduction of halogen groups to the polymer backbone proved to be detrimental for the thermoelectric behavior of the material. The miscibility of conjugated polymers and dopants are crucial for tuning the morphology and doping efficiency of polymer thermoelectric devices [72]. Kiefer et al. [73] exploited the good miscibility between the n-type naphthalenediimide copolymers and the n-dopant N-DMBI, leading to relatively high electrical conductivity (0.1 S/cm) at a low doping concentration (10%). The introduction of the dopant modified the thin film morphology, as proven from GIWAXS patterns (Figure 10(b)), resulting in a trend between power factor and electrical conductivity (Figure 10(c)) that resembles that of p-type thermoelectrics.

**Figure 11.**
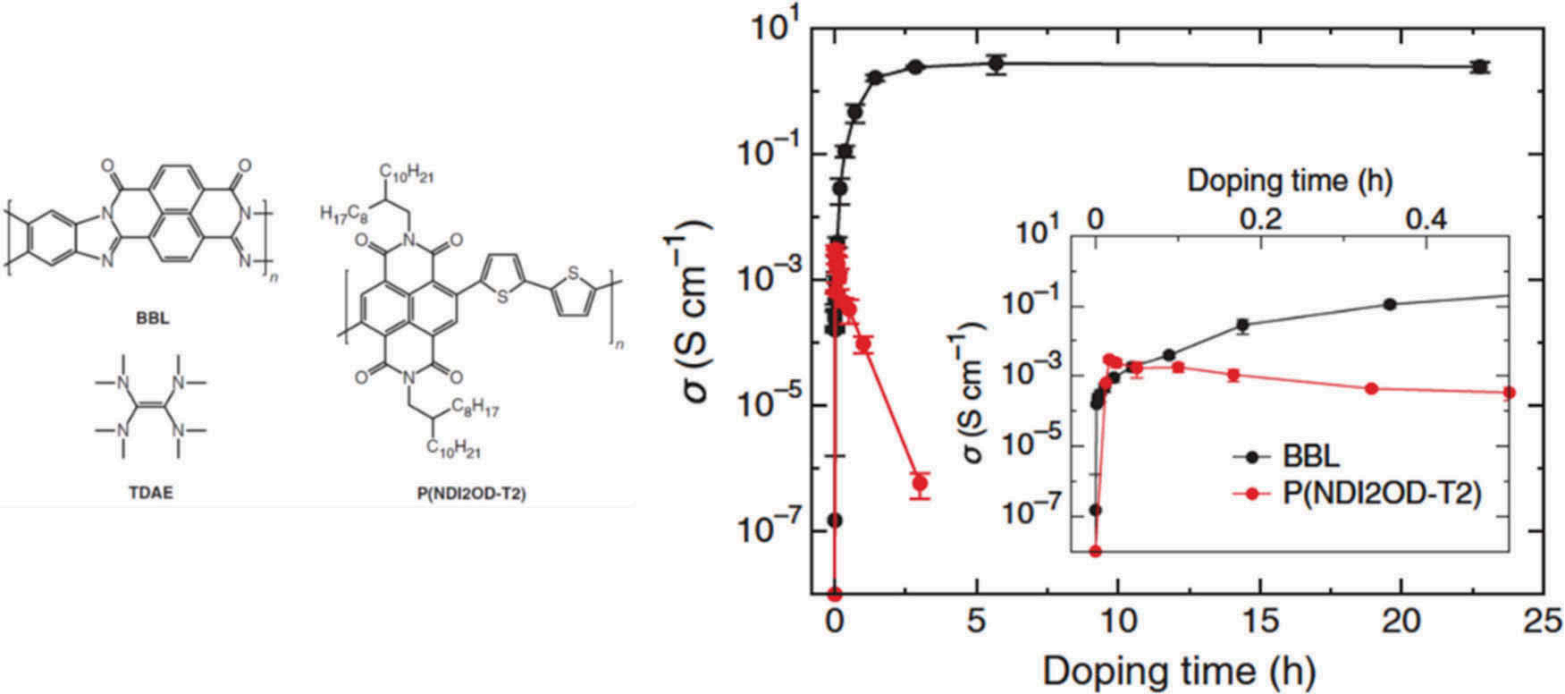
(left) The BBL, P(NDI2OD-T2) and TDAE structures, (right) the tuning of the electrical conductivity of such systems with doping time. Reprinted by permission from Wiley [74] Copyright 2017.

The thermoelectric properties of a soluble and air-stable ladder-type conducting polymer were reported in 2016 by Wang et al. [74]. The n-type polybenzimidazobenzophenanthroline (BBL) was doped with TDAE to reach an electrical conductivity of 2.4 S/cm. The thermoelectric properties of the system were optimized by tuning the doping levels through exposure of the material to TDAE (Figure 11). An optimal power factor of 0.46 μW/m∙K^2^ was achieved for BBL, one order of magnitude higher than for other soluble n-type thermoelectric polymers. In another study [75], tetrabutylammonium fluoride (TBAF) was used as a dopant for the polymer ClBPPV. The material exhibited a relatively high electrical conductivity of 0.62 S/cm and a power factor of 0.63 μW/m∙K^2^, at a 25% TBAF concentration.

### 2.5. Thermal conductivity of thermoelectric polymers

**Figure 12.**
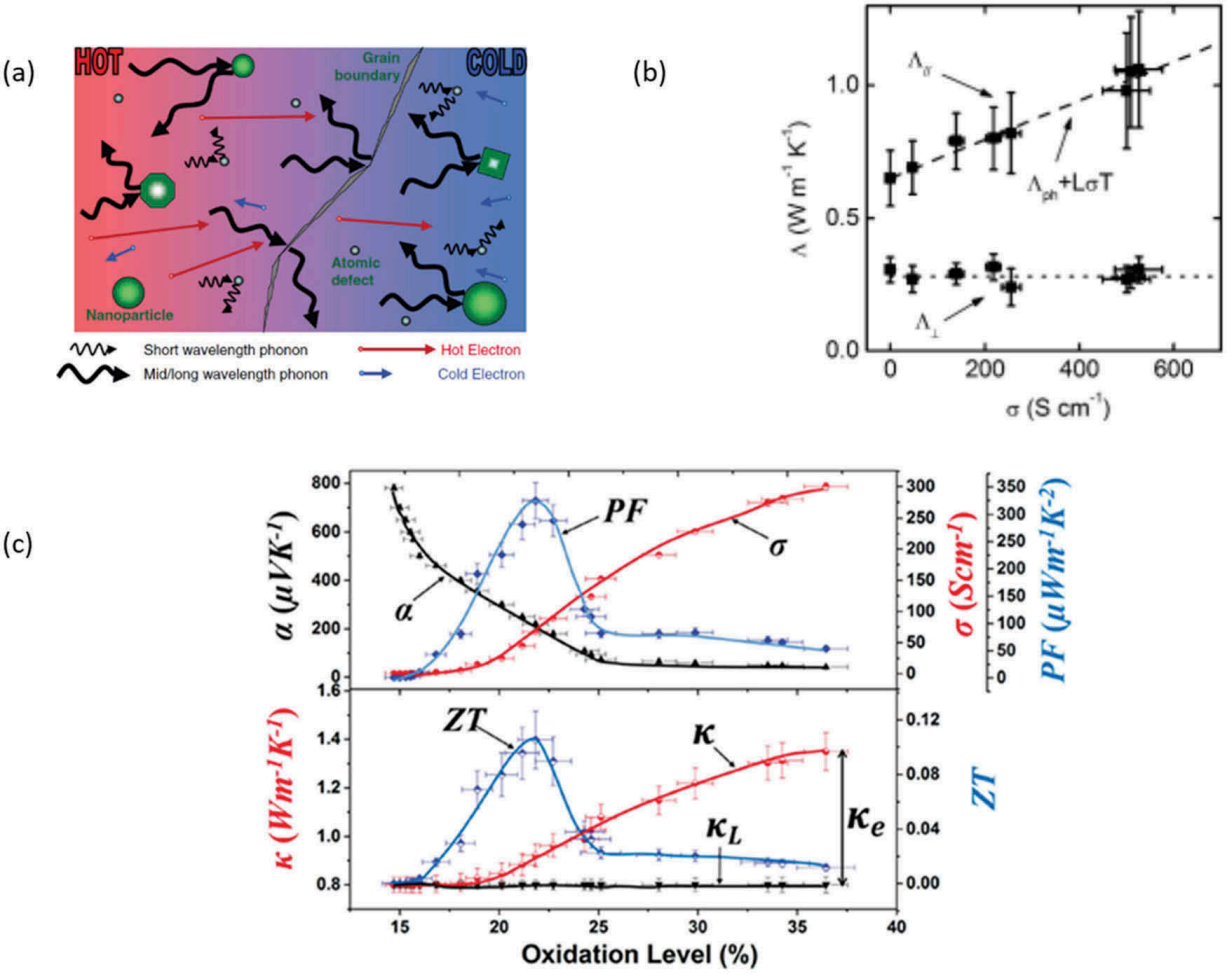
(a) A schematic description of the electron-crystal/phonon glass concept. Reprinted by permission from Wiley [76] Copyright 2010. (b) The behavior between the thermal conductivity in the in-plane and out-of-plane direction (noted here as Λ), and the electrical conductivity for PEDOT:PSS, as reported by Liu et al. [79]. The dashed line represents the Wiedemann–Franz law. Reprinted with permission from [79], Copyright 2015 American Chemical Society. (c) All the parameters necessary for the thermoelectric optimization, plotted vs the Oxidation levels for PEDOT. Reprinted by permission from Wiley [77], Copyright 2016.

In inorganic systems, a thermoelectric material is considered efficient if it satisfies the concept of electron-crystal/phonon-glass (Figure 12(a)). The material should transport electronic charge carriers (thus having high electrical conductivity, *σ*), and inhibit the diffusion of phonons, i.e. the heat carriers, thus having a low thermal conductivity (*κ)*. The total thermal conductivity of semiconducting materials is the sum of an electronic contribution *κ_e_* (charge carriers) and a lattice contribution *κ_L_* (phonons). In fact, *κ_e_* and electrical conductivity are linked through the Wiedemann–Franz law that dictates that *κ_e_* = (*k*_B_/*e*)^2^*LTσ*, where *L* is the Lorenz number. Therefore, the typical thermoelectric optimization through tuning of the doping levels is not trivial for inorganics; one should consider not only the antagonistic behavior between *S* and *σ*, but also the changes in *κ* (Figure 12(c)) [14].

Polymers provide a great advantage with respect to inorganics, as they have an inherent relatively low lattice thermal conductivity (~ 0.01–2 Wm^−1^K^−1^), which is explained from their typically amorphous morphology and the weak interchain interactions (non-covalent). Indeed, in an amorphous or semicrystalline material, the phonons encounter obstacles and scattering during their diffusion, which leads to a lower *κ_L_* (and thus *κ*) in comparison to purely crystalline systems. In doped conducting polymers, *κ_e_* is not negligible, as charge carriers can also contribute to the propagation of heat. In the limit of low electrical conductivity, the thermal conductivity for PEDOT:Tos free standing thin films approaches about 0.5 W/m∙K, which represents an estimate of the lattice contribution *κ_L_* to the thermal conductivity. Specifically, the measured thermal conductivity increases with the electrical conductivity, by as much as a factor of three when the electrical conductivity is increased from 20 to about 500 S/cm. Such an increase should not be attributed alone to the variation of *κ_L_* in those various samples, and must have an electronic origin due to the contribution of the charge carriers. However, this behavior still deviates from that of an ideal metal. An ideal metal would have a Lorenz number equal to the Sommerfeld value, 2.45 × 10^−8^ WΩ/Κ^2^, however those systems had a larger L ~ 10^−7^ WΩ/Κ^2^, which might be attributed to the inelastic scattering of the carriers in the system (due to its amorphous nature) and to the bipolar contribution of the charge carriers [78].

Other reports on the thermal conductivity of conducting polymers have also given values around 1–2 W/m∙K. Liu et al. [79] used time-domain thermoreflectance in combination with measurements of elastic constants to track down the in-plane thermal conductivity of PEDOT:PSS films and evaluated it to 1 W/m∙K. Additionally, the authors reported a *κ* vs *σ* behavior (Figure 12(b)), leading to a Lorenz number for PEDOT:PSS close to the ideal Sommerfeld value. Meanwhile, Wei et al. [80,81] determined the in-plane thermal conductivity of PEDOT:PSS films by measuring the thermal diffusivity of the material, extracting a value of 0.8 W/m∙K for PEDOT:PSS. Moreover, as Shi et al. [82] and Genovese et al. [83] underlined in their theoretical works, a more structured PEDOT film will have a higher thermal conductivity; nevertheless, upon proper optimization of the morphology, a figure of merit as high as 0.48 can be potentially reached at room temperature.

### 2.6. Polymer thermoelectric devices

**Figure 13.**
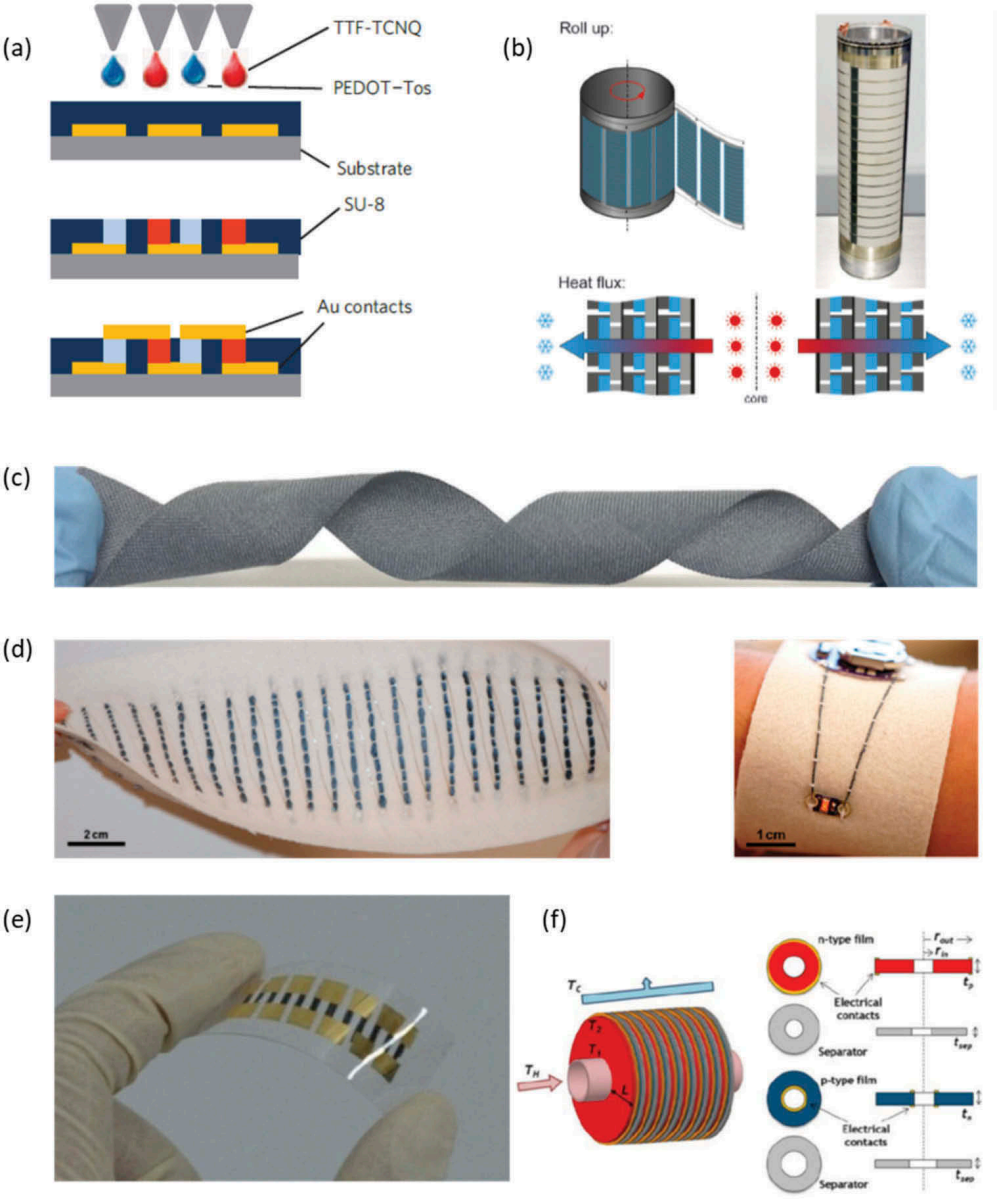
(a) The polymer thermogenerator with bulk compartments reported by Bubnova et al. [26] Reprinted by permission from Springer: Nature Materials [26], Copyright 2011. (b) Top: The thermoelectric device fabricated with the roll-to-roll process. Bottom: The heat transport for the functionality of the thermoelectric device. Reprinted by permission from [84] Wiley. Copyright 2013. (c) The polyester fabric coated with PEDOT:PSS reported by Du et al. [92]. Reprinted by permission from Springer: Scientific Reports [92]. Copyright 2015. (d) (left) The 26 p-type legs thermoelectric device with the yarn; (right) image of an LED connected with PEDOT:PSS dyed silk yarns to a battery (embroidered on felted wool fabric). Reprinted (adapted) with permission from [93]. Copyright 2017 American Chemical Society. (e) The ink-jet printed device with the metal coordination polymer, reported by Jiao et al. [95]. Republished with permission of Royal Society, from [95]; permission conveyed through Copyright Clearance Center, Inc. (f) Illustration of the radial thermoelectric generator. Reprinted (adapted) with permission from [90]. Copyright 2016 American Chemical Society.

Thermoelectric devices can be fabricated in vertical (bulk) or in lateral (thin film form). The first thermoelectric device that utilized polymers as its active elements was reported by Bubnova et al. [26] in 2011. The authors used PEDOT:Tos as the p-type thermoelectric leg and TTF-TCNQ as the n-type thermoelectric leg (Figure 13(a)). With that bulk configuration the authors managed to fabricate the first organic thermoelectric module that produced 0.128 μW with ΔΤ = 10 Κ at room temperature. More studies have appeared in the later years, trying to utilize polymers for thermoelectric devices. However, due to the much lower efficiency and stability of n-type polymers, most reported devices used only p-type thermoelectric elements with PEDOT as the active material, where the thermoelectric legs were connected in-series with a metal like silver. Such devices would have lower efficiency than a p-type/n-type configuration, but they compensated for this by increasing the number of legs and optimizing the device architecture. Sondergaard et al. [84] reported a roll-to-roll printed polymer thermoelectric device with 18,000 serially connected junctions as seen in Figure 13(b). The authors estimated that this configuration would give out 0.2 W/m^2^ of power in a payback time of 1 year. Screen printed thermoelectric devices on paper were reported by Wei et al. [85], using PEDOT:PSS and silver connections. Those devices had a power output of ~ 4 μW in a ΔΤ = 50 K, while the authors underlined that polymer thermoelectrics should be utilized for room temperature applications. In a later study [86], a thermoelectric device was fabricated by thermal lamination at 373 K, using PEDOT:PSS. In such architecture, the device exhibited a power output of 37 μW in a temperature difference of 50 K, at room temperature. The importance of device architecture was further explored by Aranguren et al. [87] in 2016. The authors reported a printed polymer π-shaped module and also developed a model to simulate, predict and optimize the thermoelectric device. Particularly, the power production of the optimized device was increased 50 times, while it was underlined that up to 21 MWh/year can be produced by such setups. Lee et al. [88] investigated the effect of aniline to the properties of a PEDOT based thermoelectric device, while they compared the power output of a vertical (drop-casted, thick film) and a horizontal (spin-casted, thin film) device. Aniline could actually control the acidity of PEDOT:PSS and enhance the performance of the thermoelectric device. Meanwhile, the authors highlighted that vertical thermoelectric devices had up to 7 times higher power (~ 175 nW @ ΔΤ = 50 Κ). Also, Gordiz et al. [89] discussed that a large number of thermoelectric legs would result in high interconnect resistance that would eventually limit the device output. They proposed that by positioning the thermoelectric legs in a hexagonal closed-packed layout, higher fill factors can be achieved that lower the total interconnect resistance and lead to higher power outputs.

An alternative to the polymer thermoelectric device architecture was proposed by Menon et al. [90,91]. In particular, they fabricated a radial thermoelectric device (Figure 13(f)), which accommodates a fluid as a heat source and can operate under natural heat convection. With such configuration the authors managed to produce 85 mV open circuit voltage and a power density of 15 nW/cm^2^. An applicable approach towards polymer thermoelectric devices was reported by Du et al. [92] and Ryan et al. [93]. In the first study, the authors coated commercial fabrics with PEDOT:PSS and used fine metal wires to connect the thermoelectric legs (Figure 13(c)). Such wearable devices generated a voltage output of 4.3 mV at a ΔΤ ~ 75 Κ. Meanwhile, Ryan et al. coated silk yarn with PEDOT:PSS (Figure 13(d)) and fabricated a washable thermoelectric wearable device with 26 legs, that gave an output voltage of 35 mV at a temperature difference of 66 K. Li et al. [94] highlighted the importance of polymer structure to the performance of a polymer thermoelectric device. In fact, by controlling the structure of free standing PEDOT:PSS films, the authors reported a 5-leg module with thermovoltage of 2 mV at a temperature difference of 25 K.

Sun et al. [65] fabricated vertical thermoelectric modules using p-type and n-type metal coordination polymers. In fact, the fabricated devices with only 35 thermocouples produced a thermovoltage of 0.25 V with a temperature difference of 82 K, and a power output per area ~ 3 μW/cm^2^ with ΔΤ = 30 Κ. Those devices were made printable by Jiao et al. [95] by mixing the active material with polyvinylidene fluoride (PVDF) solutions. A flexible device of only 6 thermocouples (Figure 13(e)) would produce a voltage of 15 mV at a ΔΤ = 25 Κ. Finally, Liu et al. [68], fabricated an n-type-only thermoelectric module based on a metal coordination polymer with an electrochemical deposition process. The authors argued that with this technique a device with 108 legs can be fabricated, while they demonstrated that a simple device of 18 legs can produce an output power of ~ 0.5 μW with only a temperature difference of 12 K and a power density as high as ~ 580 μW/cm^2^.

Besides energy harvesting, thermoelectric devices can be also used as temperature sensors. Taroni et al. [96] fabricated blends of conducting PEDOT:PSS with the stretchable, but insulating, polyurethane. Although the power factor of the system was decreasing with % of the polyurethane, those mixtures enabled the fabrication of self-powered motion sensors, highlighting the applicability of thermoelectric polymers. A novel approach utilizing the polymer morphology for thermoelectrics is the fabrication of aerogels. Gordon et al. [97] had fabricated soft and light-weight PEDOT:PSS aerogel architectures by freeze-drying. Although such systems had a low power factor (~ 7 μW/m∙K^2^), they are perspective materials for pressure and temperature sensors. That was the case in the work of Han et al. [98], with PEDOT:PSS/nanocellulose aerogels. Such systems had a semimetallic behavior and were used for the fabrication of functional temperature/pressure sensors.

In view of possible compatibility with general semiconductor fabrication methods, a photolithography method was used to fabricate a π-type flexible organic module [99]. PEDOT:PSS and TTF-TCNQ were used as the p- and n-type materials, respectively. An output voltage of 250 mV, sufficient to drive electrical devices with a booster circuit, was realized at 80 K temperature difference. However, the high contact resistance was a problem, and it was proposed to design thermoelectric materials from the standpoints of expected module structures and mass-production processes rather than optimize ZT of the material first [99].

## 3. Organic–inorganic hybrid thermoelectrics

Organic–inorganic hybrid thermoelectrics (TEs) are a fascinating field for both organic and inorganic materials researchers. Although Shirakawa et al. discovered conductive organic polymers [100], most stable organic TE materials are p-type materials and they have lower *ZT* than inorganic TE materials. Hybridizing with inorganic TE materials is another pathway to enhance the TE performances and a promising way to acquire n-type properties. For inorganic TE materials, hybridizing with organic materials provides flexibility and low thermal conductivity: advantages in energy harvesting. Because of these reasons, organic–inorganic hybrid TEs have been actively investigated [101].

**Figure 14.**
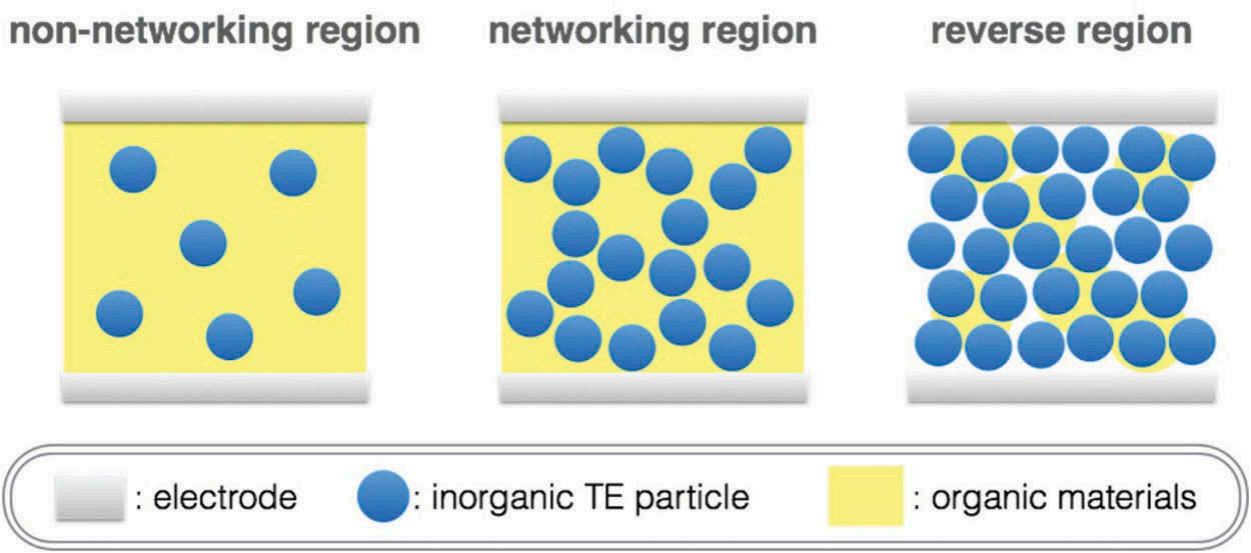
Schematic image of organic–inorganic hybrid TE materials depending on the volume ratio.

Recently, there have been notable studies on composite thermoelectric materials in general. Initially for some inorganic systems, enhancement of the power factors through mechanisms like energy filtering [102] or modulation doping [103] have been proposed. There has also been enhancement observed for highly conducting metallic networks doped into ceramic borides, for example [104]. To get a simple general picture of organic–inorganic hybrid TEs without including any such exotic effects, we first suppose a certain size of spherical inorganic TE particles mixed with organic materials (Figure 14). It can be assumed that we can obtain the generated TE power from the inorganic TE particles when the inorganic TE particles network for electric conduction. Herein, we can classify the volume ratio of inorganic TE particles into three regions: (1) non-networking region for the low volume ratio, (2) networking region for the middle volume ratio, and (3) reverse region for the high volume ratio. In the non-network region, it can be expected that the inorganic TE particles do not assist the TE performances. In contrast, as far as we assume spherical particles, the reverse region is not our interest because the inorganic TE materials do not obtain flexibility. Thus, the hybrid effect should be observed in the network-forming region. The maximum ratio for the network-forming region is around 74 % where the spherical particles give the closest packing fulfilled with the organic materials. In connection with the relation between the volume ratio and the packing style, the body-centered cubic packing, the primitive cubic packing, and the diamond cubic packing develop at the ratio of 68 %, 52 %, and 34 %, respectively. It suggests that the inorganic TE particles have the potential to output generated power in the wide volume ratio from 34 % to 74%. To optimize the TE performances, we need to choose the appropriate particle sizes to utilize the mean free paths of electron and phonon because the particle network for electric conduction can also conduct heat. In similar systems, such as electrically conductive adhesives; metal-resin composites, and thermal conductive sheets; filler-silicone composites, percolation theory explains electric and thermal conductions, where the interfacial resistances play the dominant role [105,106].

Secondly, we review experimental reports of the organic–inorganic hybrid TE materials. Bi_2_Te_3_-type materials are inorganic TE materials generally recognized to exhibit high *ZT* near room temperature. Related to the interest in possible quantized effects on the Seebeck coefficient *S* [107,108], 5-nm, 20-nm, and 2-µm Bi_2_Te_3_ particles have been hybridized with polyaniline [109]. Although the smallest particles are reported to show enhancement of *S*, from 17.9 µV/K to 120 µV/K, electric conductivity σ maintains or decreases from 37 S/cm to 2 S/cm, depending on the mixing condition. This supports the idea of the importance to control the interface for electric conduction. To solve the problem, various additives and high-temperature annealing with thermal-resistive additives have been examined to achieve equivalent thermoelectric performances with bulk Bi_2_Te_3_ (thermoelectric figure of merit, *ZT* = 0.97) via recovering the disadvantage of low *σ* by the reduced thermal conductivity [110–113]. Instead of surface-sensitive TE materials, carbon nanotubes have been also investigated actively in composites, because carbon nanotubes have self-passivated surfaces to reportedly achieve 2 orders of magnitude higher TE performances than just the original organic materials [114–116]. Basically, the above results can be discussed from a macro-scale viewpoint based on percolation theory.

Another interesting approach for organic–inorganic hybrid TEs is to utilize molecular intercalation [8]. Wan et al. discovered that intercalating organic molecules resulted in making n-type inorganic TiS_2_ flexible and less thermally conductive [117,118]. Additionally, the carrier mobility is affected by the dielectric constant of the intercalation molecules [119]. As a result, the *ZT* value for this n-type flexible material reaches 0.33 at 413 K, which is significantly higher than the original TiS_2_ single crystal. Prototype modules with organic p-type and using this type of TiS_2_ hybrid material as the n-type have also been constructed with relatively high power densities [8]. Overall, the hybrid TEs are opening a notable possibility especially for n-type TE materials toward energy harvesting.

Recently, further efforts have been made to control and utilize the interfaces of the organic and inorganic materials for enhancing thermoelectric properties. Chen and coworkers focused on the role of surfactants during the mixing procedure [120]. They note that in many typical cases thermoelectric enhancement in organic–inorganic hybrid materials has not been observed, and attribute that to phase separation and inhomogeneous mixing in the composite. This is slightly related to what was discussed above in Figure 14, where the minimum requirement is to have enough volume of inorganic material to create effective connections. However, even having sufficient amount of inorganic materials, if the mixing is poor resulting in inhomogeneity, then benefits from the hybridization will not be realized. Types and concentrations of surfactants were altered to systematically change the homogeneity of the mixture of PEDOT:PSS and Bi_0.5_Sb_1.5_Te_3_. It was shown that C_14_H_22_O(C_2_H_4_O)_n_ with n = 9 or 10 (TX-100) which has a hydrophobic tail is more effective at creating a randomized mixing than often used dimethyl sulfoxide (DMSO), leading to an enhancement in both Seebeck coefficient and electrical conductivity. This result underlines the importance to achieve a homogeneous mixing in such hybrid materials [120].

In regards to the quality of the interface between the organic and inorganic materials, a detailed theoretical investigation was carried out by Malen et al. on the thermoelectric transport of a junction of a single organic molecule with inorganic contacts [121]. They point out that the sharp peaks in the density of states at the Fermi level are similar to the ideal *ZT* case of the electronic transport through a single energy level demonstrated by Mahan and Sofo [122]. Using the Lorentzian transmission model, the *ZT* of the *i*th molecular orbital, *Z_i_T*, can be approximated as follows:

(1) $$Z_{i}T\approx 4p^{2}{k_{B}}^{2}T^{2}/3(\mu -E_{i})2\left(1+R\right)$$

where *E_i_* is the energy level of the *i*th molecular orbital, μ is the chemical potential, and *R* is the ratio of the phonon thermal conductance to the electron thermal conductance. Therefore, when the energy levels of the molecular orbitals have good alignment with the chemical potential of the inorganic contacts, the *ZT* can be optimized [121]. Furthermore, the phononic heat transport through the interfaces can be expected to be low, because of the large difference in vibrational spectra between the organic molecules and inorganic bulk [121]. Such interfaces are potentially useful if they can be controlled. Since inorganic contacts, i.e. electrode materials typically do not have such low Fermi levels, energy level matching is difficult in practice, and the same authors therefore, propose utilizing the lowest unoccupied molecular orbital (LUMO) [123]. They observe by varying the metal contact and lowering the work function, that the Seebeck coefficients of C_60_, [6,6]-phenyl-C_61_-butyric acid methyl ester (PCBM), and C_70_ can be monotonically increased to n-type absolute values above 25 μV/K [123].

The energy filtering effect at the organic–inorganic interfaces have also been reported to enhance the thermoelectric properties, for example, in poly(3-hexylthiophene) (P3HT) where nanocomposites were formed with Bi_2_Te_3_ nanowires [124]. The interfacial barrier height range to effectively scatter low energy carriers under high electrical conductivity is considered to be 0.04–0.10 eV [125]. In the energy filtering scheme, low energy carriers are scattered at the interfaces, with high energy carriers which can carry more heat transporting across and thereby enhancing the Seebeck coefficient [102]. P3HT was doped with FeCl_3_ to tune the electrical conductivity, with heavy doping leading to an interfacial barrier height below 0.10 eV in the energy band diagram, matching the region where enhancement of the power factor of the nanocomposite was observed. P3HT-Bi_2_Te_3_ nanocomposites formed using Bi_2_Te_3_ nanoparticles did not show enhancement which was speculated as due to the low energy electron wave functions being able to go around the nanoparticles [124].

To summarize, the careful control and utilization of the interface between the organic and inorganic materials, is demonstrated to be a route which can provide substantial enhancement of the thermoelectric properties in the hybrid materials.

Finally, various thermoelectric modules have been fabricated using organic–inorganic hybrid materials, due to the advantages of flexibility that are conferred by the organic material. For example, hybrid nanocomposite PEDOT:PSS film structures with Bi_2_Te_3_ or Sb_2_Te_3_ nanocrystals as the inorganic material were print-fabricated on polyimide sheets, using an aerosol jet printing method. The power output wasn’t determined, although the thermoelectric properties were evaluated as having the maximum performance at 85 wt% of Sb_2_Te_3_, with *S* ∼33.8 μV/K, *σ* ∼247.3 S/cm, and power factor, *S^2^σT* ∼28.3 μW/m∙K^2^ [126]. In a slightly unusual device structure, flexible transparent modules were reported, by using polyethylene terephthalate (PET) substrate onto which, PEDOT:PSS and a hybrid indium tin oxide (ITO)-PEDOT:PSS material were deposited as the p- and n-type legs, respectively. From 8 p-n pairs, a voltage output of 6.8 mV and power output of 0.86 nW are generated with a temperature difference of around 20 K. The n-type thermoelectric properties appear dominated by the ITO with no enhancement from the hybrid effect, however, the authors propose that the PEDOT:PSS coating on top of ITO promotes mechanical stability during the active bending of the module [127].

Koumoto and coworkers have fabricated a thermoelectric module composed of hybrid TiS_2_/organic superlattice films and PEDOT:PSS film, as the n- and p-type materials, respectively. From 5 p-n pairs, they obtain a voltage output of 33 mV with a maximum power density of 250 μW/cm^2^ under a temperature difference of 70 K [127,128].

## 4. Thermoelectric inorganic film: flexible thermoelectric device and micro-thermoelectric generator

**Figure 15.**
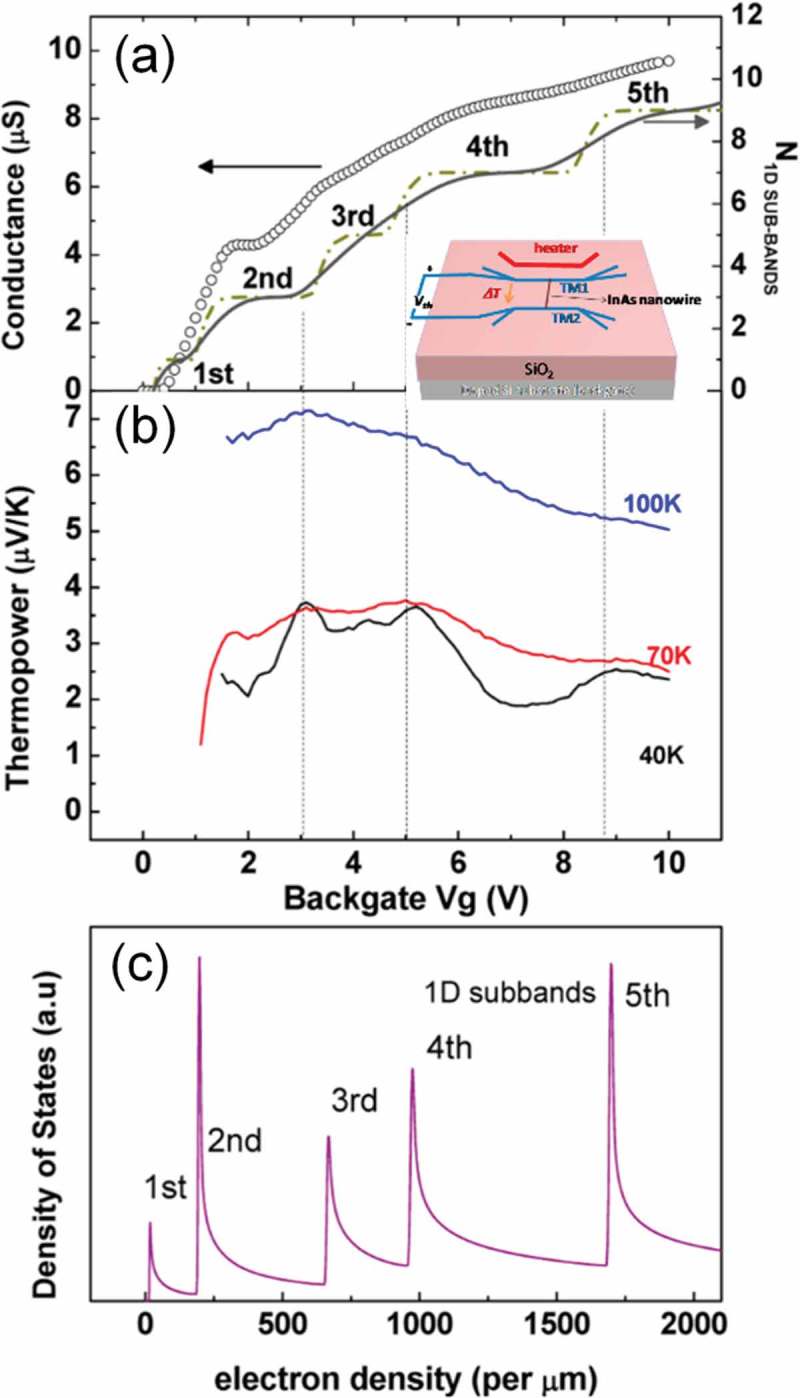
Gate-tuned conductance and thermopower of InAs nanowire. (a) Comparing the measured conductance (40 K) vs gate voltage (V_g_) data (open circle) with the calculated one dimensional (1D) subband occupation with only thermal broadening (dash dotted line) or both the thermal and the scattering broadening (solid line) considered. (b) Gate modulation of thermopower (S) at 100, 70, and 40 K. The dashed vertical lines are a guide to the eye, highlighting the appearance of peak in S(V_g_) when a 1D subband starts to be filled. (c) Calculated density of states vs 1D electron density in nanowire with the index of subbands marked. Reproduced with permissions from [135].

Theoretical work to improve the thermoelectric conversion efficiency owing to quantum effects appearing in low-dimensional structures such as superlattices, proposed by Dresselhaus et al. [106], motivates thin-film researchers to investigate thermoelectric materials. The use of quantum-confinement phenomena enhances Seebeck coefficient (S) and control Seebeck coefficient and electrical conductivity (σ) somewhat independently. Phonon scattering becomes more effectively induced by numerous interfaces, resulting in lower thermal conductivity. The thermoelectric properties of superlattices based on various materials have been investigated. Enhanced Seebeck coefficient and power factor (*S^2^*σ) is demonstrated using (001) oriented Si/Ge superlattices reported by Koga et al. [129]. Venkatasubramanian et al. reported figure of merit (ZT) enhancement in superlattices composed of Bi_2_Te_3_ and Sb_2_Te_3_ layers, which are well-known high performance thermoelectric materials [130]. Reduction of lattice thermal conductivity in PbTe/PbTe_0.75_Se_0.25_ superlattices was found by Caylor et al. [131]. There are some oxides with non-toxic elements that are promising for use as thermoelectric materials. Ohta et al. demonstrated the enhancement of Seebeck coefficient in SrTiO_3_/Sr(Ti,Nb)O_3_ superlattices [132]. The thermoelectric properties of one-dimensional nanowires have also been studied [133]. One dimensional quantum confinement offers sharper density of states (DOS) of electrons than higher dimensional quantum confinements. Seebeck coefficient can be enhanced due to the sharper DOS [134]. The quantum confinement effects have not been confirmed experimentally, except maybe for a few works. Tian et al. fabricated InAs nanowires by chemical vapor deposition (CVD), and observed oscillations in the Seebeck coefficient and power factor concomitant with the stepwise conductance increases due to the one dimensional quantum confinements in InAs nanowires (Figure 15) [135].

Thin-film thermoelectric materials tend to have much reduced lattice thermal conductivity and thus, they exhibit enhanced thermoelectric performance compared with that of bulk materials owing to surface and interface scattering of phonons [130,131,136]. Takashiri et al. carefully investigated the influence of grain size on thermal conductivity in polycrystalline bismuth telluride based thin films [136]. The lattice thermal conductivity decreased rapidly with the decrease of the size of grains, indicating the enhancement of phonon scattering at the interfaces of grains. The total thermal conductivity of a Bi_2_Te_2.7_Se_0.3_ polycrystalline thin film with the average grain size of 30 μm was 1.6 Wm^−1^K^−1^. By decreasing the average grain size down to 10 nm, the total thermal conductivity reached 0.61 Wm^−1^K^−1^. Moreover, compared with thermoelectric bulk materials, thermoelectric devices realized via thin-film preparation offer the remarkable advantage of localized and rapid cooling owing to their capacity for miniaturization [137,138]. Recently, various techniques of film fabrication on flexible substrates such as polymer sheets and metal foils have been developed [139]. Thus, the development of flexible thermoelectric devices has been carried out extensively.

**Figure 16.**
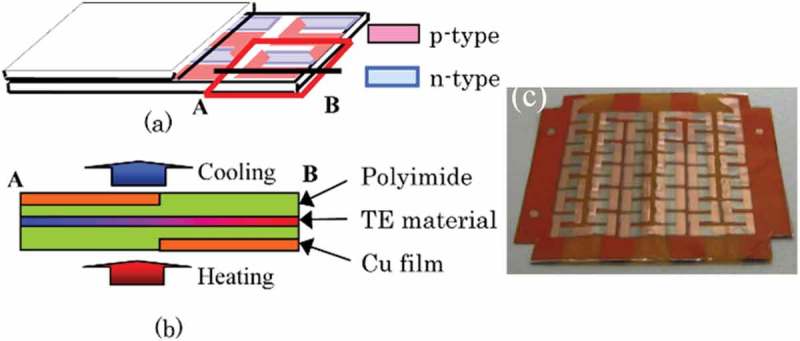
Schematic illustrations (a) (b) and picture (c) of the flexible thermoelectric (TE) device developed by Takeda et al. The flexible thermoelectric device consists of 33 pairs of p-n couples composed of chromel and constantan layers within approximately 30 mm × 30 mm. Reproduced with permissions from [140] and [141].

**Figure 17.**
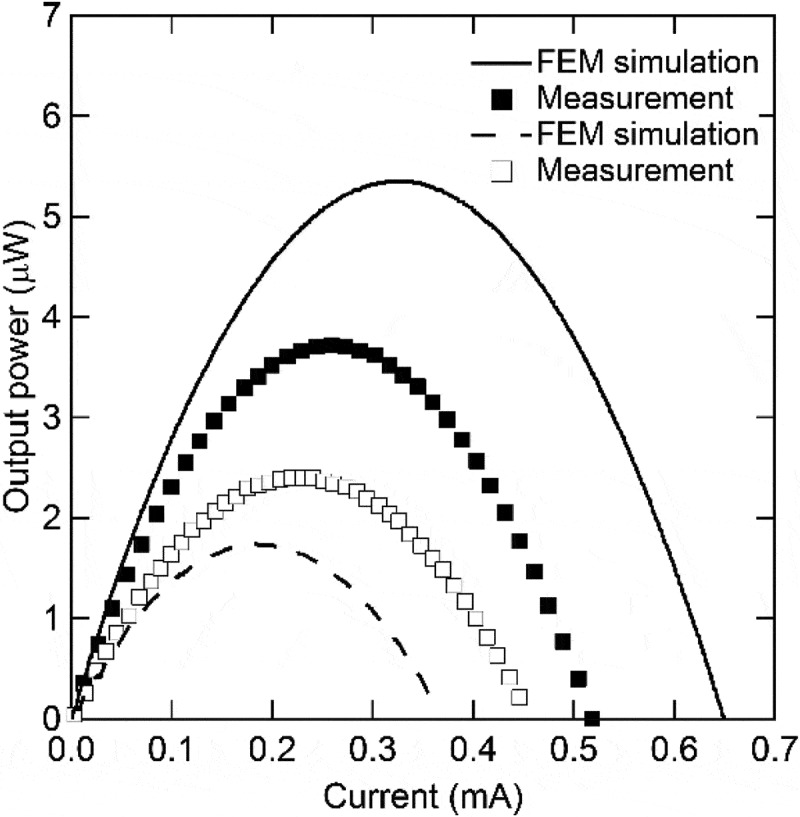
Current–output power characteristics of two different flexible thermoelectric devices reported by Takeda et al. Reproduced with permission from [141].

Takeda et al. fabricated flexible thermoelectric devices composed of chromel (90 % nickel-10 % chromium) and constantan (55 % copper-45 % nickel) layers as p- and n-type elements to form p-n couples covered with another flexible sheet (Figure 16) [140,141]. The chromel, constantan, and copper layers with thickness of approximately 1.25–2.50 μm were prepared via radio frequency (RF) magnetron sputtering on a flexible substrate composed of polyimide and copper sheets. The flexible substrate was composed of high and low thermal conductivity materials. The electrical conductivity (σ) and Seebeck coefficient (S) were 1.02 × 10^6^ Ω^−1^m^−1^ and 19.1 μV/K, respectively, for the chromel layer (thickness 1.5 μm) and 1.50 × 10^6^ Ω^−1^m^−1^ and −41.1 μV/K, respectively, for the constantan layer (thickness 2.5 μm). These values are comparable to the bulk electrical conductivity and bulk Seebeck coefficient of 1.43 × 10^6^ Ω^−1^m^−1^ and 22 μV/K, respectively, for chromel and 1.79 × 10^6^ Ω^−1^m^−1^ and −40 μV/K, respectively, for constantan. The performance of this flexible thermoelectric device was improved using the finite element method (FEM). Accordingly, these flexible thermoelectric devices generated output power of 2.40–3.72 μW from a temperature difference of 22.7–24.0 K applied between the outer surfaces of the device (Figure 17).

**Figure 18.**
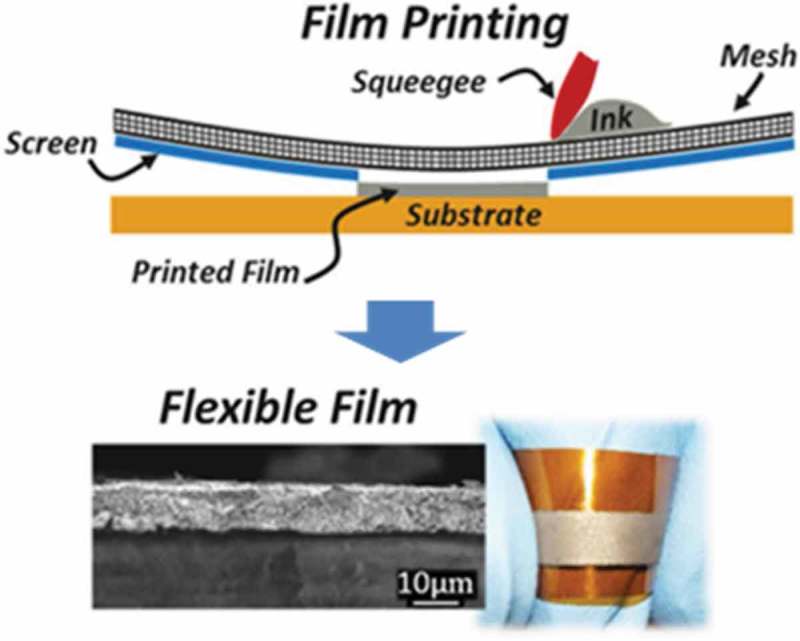
Schematic illustrations of the preparation of flexible film using screen-printing technique reported by Varghese et al. Reproduced with permissions from Ref. [142].

**Figure 19.**
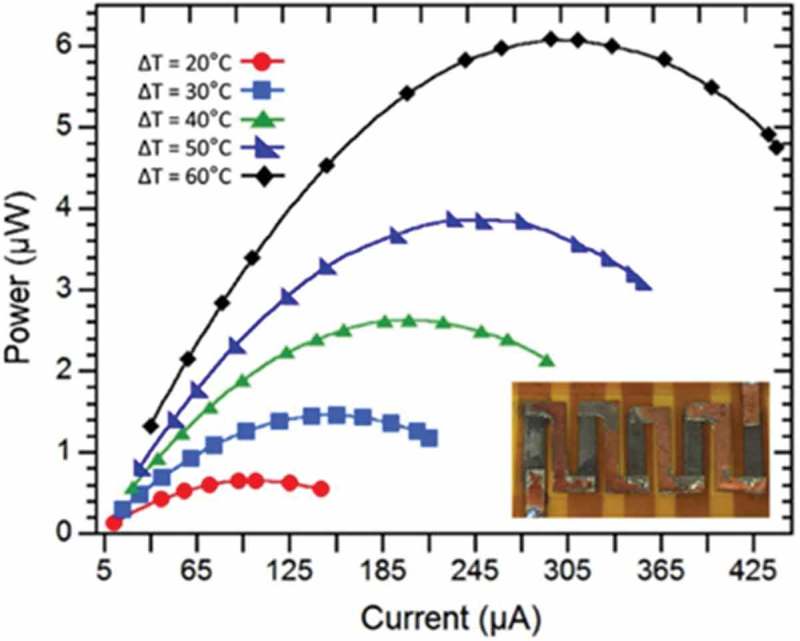
Current–output power characteristics of flexible thermoelectric devices prepared using screen-printed Bi_2_Te_2.8_Se_0.2_ film. Inset is a picture of the devices. Reproduced with permissions from [142].

Recently, thermoelectric devices fabricated using screen-printed flexible films have been reported by Varghese et al. [142]. Nanocrystal ink of doped Bi_2_Te_2.8_Se_0.2_ was synthesized using a microwave-stimulated wet-chemical synthesis method based on inexpensive organic solvents and metal salts (Figure 18). The as-prepared nanocrystal ink was screen printed on flexible polyimide substrates. The film thickness could be controlled to be in the range of 10–100 μm using the screen mesh size. The electrical conductivity and Seebeck coefficient of Bi_2_Te_2.8_Se_0.2_ were approximately 2–3 × 10^4^ Ω^−1^m^−1^ and −140 μV/K, respectively, at 473 K. The thermal conductivity was approximately 0.6 Wm^−1^K^−1^, which is very low in comparison with that of a Bi_2_Te_2.8_Se_0.2_ pellet. Thus, the dimensionless figure of merit, ZT, reached 0.43 at 448 K. As shown in Figure 19, the thermoelectric device fabricated using a screen-printed flexible Bi_2_Te_2.8_Se_0.2_ film generated a maximum output power of 6.1 μW from a temperature difference of 60 K.

**Figure 20.**
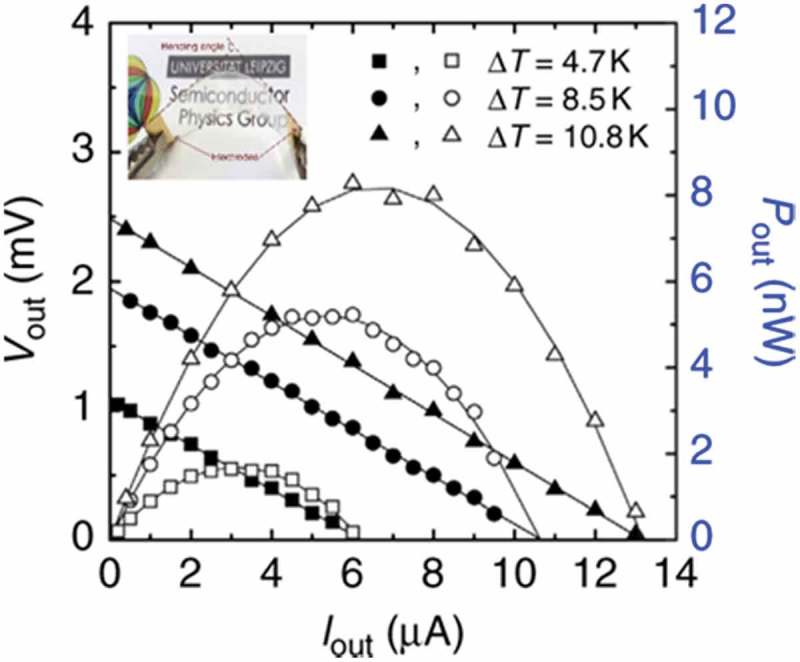
Output voltage (V_out_) and output power (P_out_) of a γ-CuI-based flexible thermoelectric device. Inset is a picture of the transparent γ-CuI film on flexible PET substrate. Reproduced with permissions from [143].

Another interesting example, a transparent flexible thermoelectric device fabricated using γ-CuI film reported by Yang et al. is shown in Figure 20 [143]. γ-CuI is a native p-type semiconductor with an indirect band gap (*E*_g_) of 3.1 eV. Polycrystalline γ-CuI films were deposited on glass and flexible polyethylene terephthalate (PET) substrates under iodine vapor condition at room temperature via reactive sputtering. At room temperature, the Seebeck coefficients of γ-CuI film on glass substrate were in the range of 150–300 μV/K in the p-type carrier concentration range of 10^19^–10^20^ cm^−3^. The maximum power factor was 3.75 × 10^−4^ Wm^−1^K^−2^. The authors reported very low in-plane thermal conductivity of 0.5–0.56 Wm^−1^K^−1^ in the γ-CuI film. The maximum *ZT* value reached 0.21 at 300 K. A transparent single thermoelectric leg of γ-CuI film was fabricated on a PET substrate. Figure 20 shows the output voltage and output power of γ-CuI-based flexible thermoelectric devices. This single thermoelectric leg generated a maximum output power of 8.2 nW from a temperature difference of 10.8 K.

**Figure 21.**
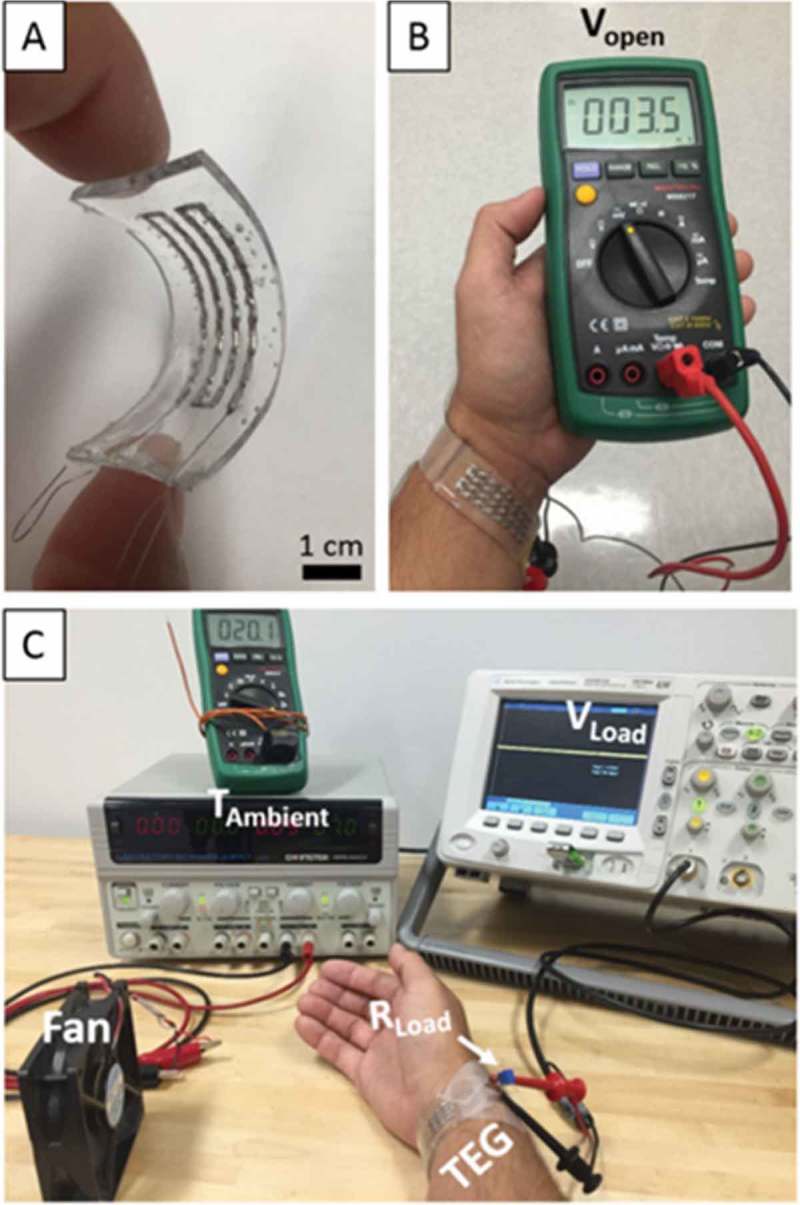
Thermoelectric generation using flexible thermoelectric devices from the human body reported by Suarez et al. (A) Flexible thermoelectric device test, (B) open circuit voltage at room temperature, and (C) test setup for thermoelectric device performance. Reprinted with permissions from [145].

Suarez et al. demonstrated thermoelectric generation from the human body using flexible thermoelectric devices (Figure 21) [144,145]. The flexible thermoelectric devices were fabricated using commercial Bi_0.5_Sb_1.5_Te_3_ p-type and Bi_2_Se_0.3_Te_2.7_ n-type legs. To improve the thermoelectric device performance, a eutectic alloy of gallium and indium was used as a stretchable low resistivity interconnector between the legs. The thermoelectric devices were designed by the modeling calculation. A system *ZT* of 0.35 at a fill factor of 6% was observed for the proof-of-concept devices. At a temperature of 297 K, the devices showed a maximum output voltage and power in the range 1.47–2.96 mV and 1.48–6.0 µW for stationary and full air velocities, respectively. The maximum temperature difference for this ambient condition was Δ*T* = 0.4 K. Thus, such flexible thermoelectric devices will pave the way for various low-temperature energy harvesting applications such as self-powered wearable electronics.

Micro-thermoelectric generators fabricated by semiconductor microfabrication techniques are also useful for energy harvesting applications in the Internet of Things (IoT) [146]. With the remarkable developments achieved in the IoT, several more devices will be included in the network for data exchange, such as wireless sensor networks (WSNs) used for environmental and building monitoring, animal tracking and control, process monitoring, security and surveillance, and medical treatment. In order to power the large amount of WSNs, suitable power sources are required. Micro-thermoelectric generators are highly suitable for energy harvesting applications due to their advantages of having high reliability, long lifetime, no moving parts, no maintenance requirements, and direct conversion with no intermediate process. Micro-thermoelectric generator technology has already been introduced in several commercial devices starting, with the Seiko Thermic watch [147].

**Figure 22.**
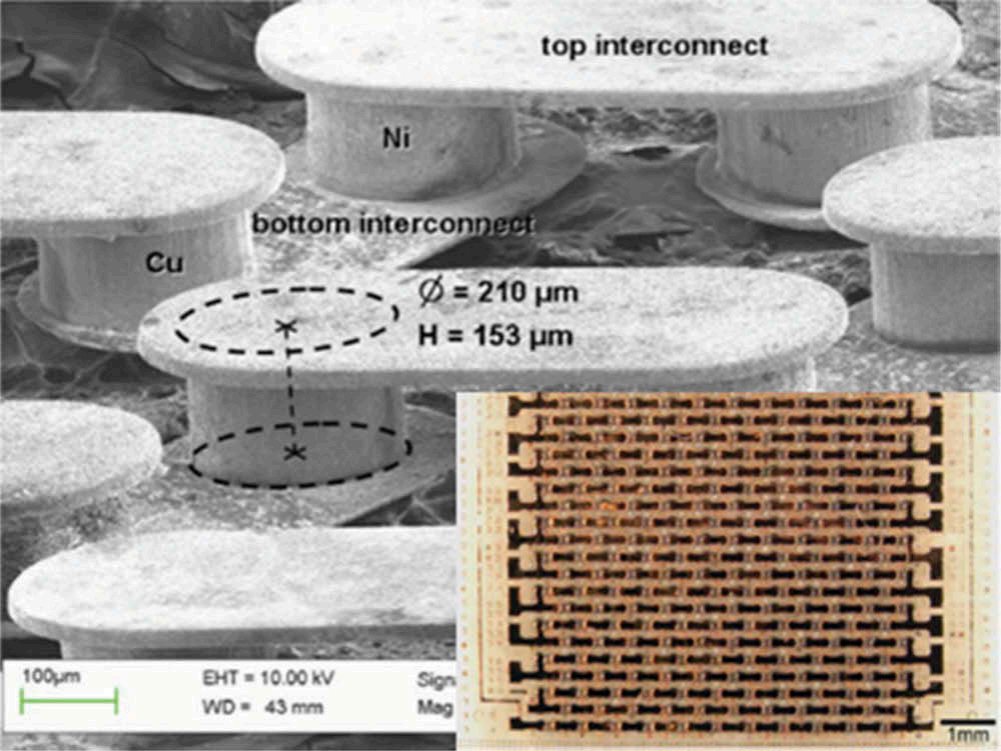
Scanning electron microscope (SEM) image of micro-thermoelectric generator composed of Ni-Cu-based thermocouples reported by Glatz et al. Inset is a picture of a micro-thermoelectric generator with 253 thermocouples. The leg diameter is 210 µm, and the thickness is 140 µm. Reprinted with permission from [148].

Micro-thermoelectric generators composed of Ni-Cu-based thermocouples were reported by Glatz et al. as shown in Figure 22. The overall system optimization was demonstrated with the help of the Ni-Cu-based micro-thermoelectric generator. The fabricated devices generated up to 2.6 × 10^−3^ μWcm^−2^K^−2^ in the planar state. The fabrication could be considered low-cost because only high rate and batch compatible micro electro mechanical systems (MEMS) standard processes were applied.

**Figure 23.**
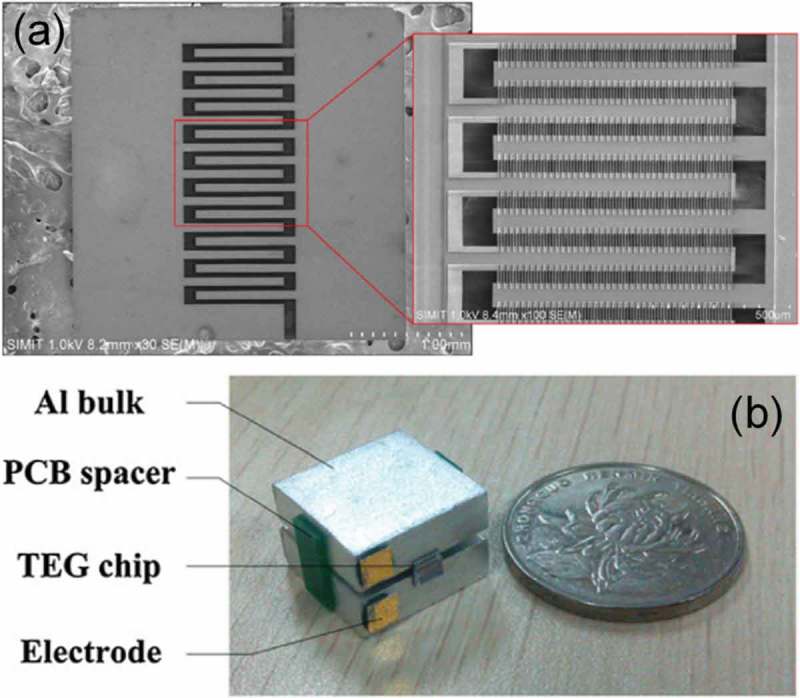
CMOS MEMS-based micro-thermoelectric generator composed of n- and p-type poly-Si reported by Yu et al. (a) SEM photograph of micro-thermoelectric generators and (b) picture of a test module. Reprinted with permission from [149].

**Figure 24.**
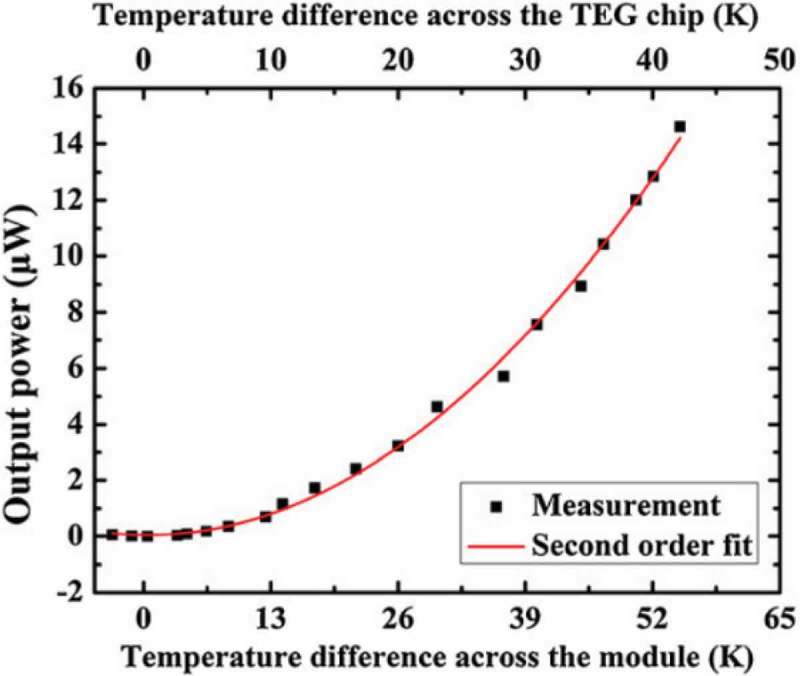
Output power versus temperature difference across the CMOS MEMS-based micro-thermoelectric generator module reported by Yu et al. Reprinted with permission from [149].

CMOS (complementary metal oxide semiconductor) MEMS-based thermoelectric generators were also developed [146,149]. Yu et al. demonstrated fully integrated micro-thermoelectric generators using n-type and p-type polysilicon (poly Si) (Figure 23). These materials were very compatible with the CMOS process and MEMS techniques with the advantages of having low cost mass production by utilizing semiconductor foundries. In order to reduce the thermal contact resistance and efficiently dissipate heat from the in-plane thermopile, a silicon substrate was etched into two comb-shaped blocks, which were thermally isolated from each other and used as the hot and cold sides of the thin-film-based legs, respectively. An individual module of micro-thermoelectric generators with a size of 3 mm × 3 mm presented an open-circuit output voltage of 146 mV/K, an output power of 14 µW, and 0.252 µWcm^−2^K^−2^ for the power factor (Figure 24).

**Figure 25.**
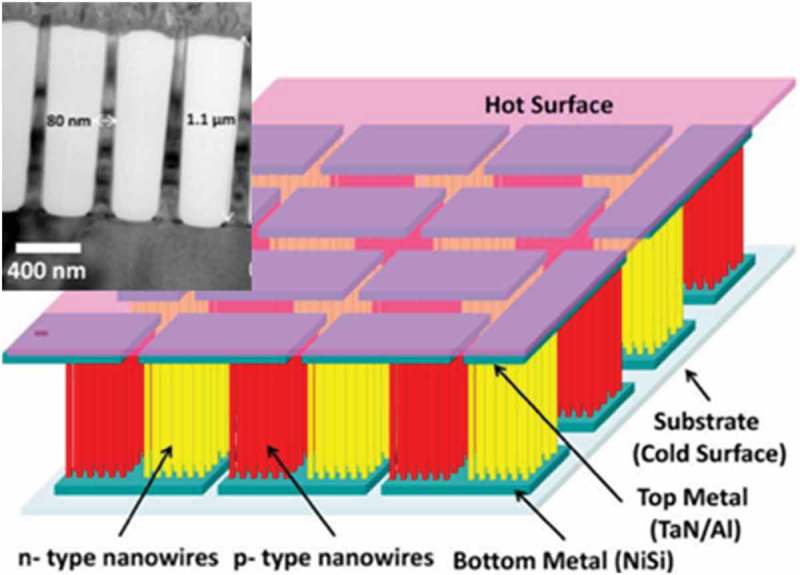
Schematic illustration and SEM image of Si nanowire micro-thermoelectric generators reported by Li et al. Reprinted with permission from [151].

**Figure 26.**
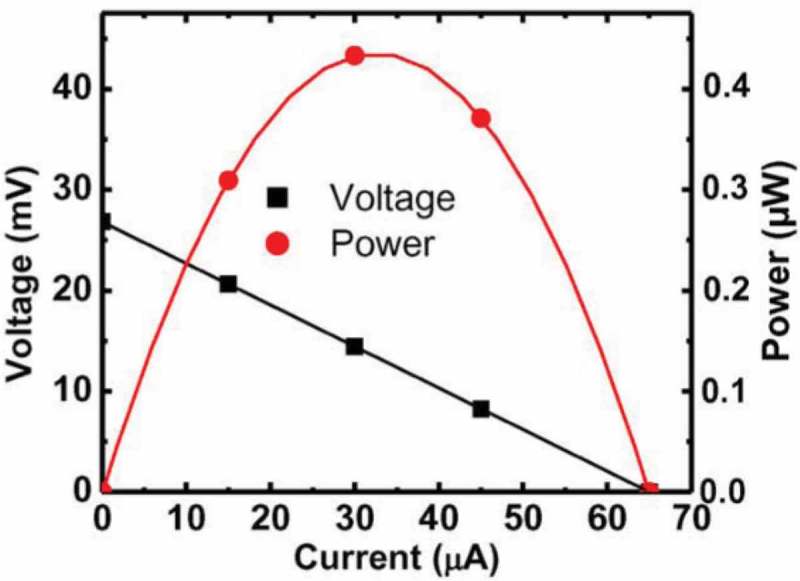
Output voltage and output power of a Si nanowire micro-thermoelectric genreators reported by Li et al. Reproduced with permission from [151].

Nanowire materials are highly suitable for application to micro-thermoelectric generators. Li et al. proposed a chip-level vertical micro-thermoelectric generator based on a high-density Si nanowire array fabricated with CMOS technology (Figure 25) [150,151]. Their device showed a maximum output power of 0.47 μW under a temperature difference of 70 K across the experimental setup (Figure 26).

As described in this section, although inorganic materials themselves are not intrinsically flexible, there have been good efforts in combination with flexible substrates, stretchable interconnects to create flexible modules. Thin film devices and micro-thermoelectric generators are also a promising technology to power WSNs in the IoT society.

## 5. Summary and outlook

Finding viable technologies to draw energy from the environment and supply it to wireless sensor networks, which will be essential to the future IoT society, is an important technological challenge. In terms of their reliability as solid state devices with no moving parts, and the ubiquitous and constant nature of heat sources including body heat, thermoelectric materials & devices can be considered to be one of the most reliable and promising energy harvesting technologies. In this review article, we have covered the fundamental strategy of thermoelectric materials development to the state-of-the-art realization of high thermoelectric figure of merit achieved in materials suitable for energy harvesting, with the foremost subject being organic thermoelectric materials. Organic–inorganic hybrid materials were also discussed, and for inorganic materials, mainly applicative endeavors were reviewed.

Many proof of concept works utilizing organic materials and inorganic materials in flexible or micro-thermoelectric generators were also described in detail. Organic thermoelectric devices have an advantage in their general lightweight, flexibility, and potentially low cost implementation/large area mass production processes such as roll to roll. The wide variety and number of organic materials and speed in which the research appears to be proceeding due to quicker synthesis processes is another attraction. The inorganic thin film devices and micro-thermoelectric generators have advantages such as potential low cost mass production by utilizing semiconductor foundries, long lifetime, and relatively high output voltage. In either case, with advantages such as no moving parts, high reliability, no maintenance requirement, flexible or small size, thermoelectric devices as energy harvesters, exhibit the potential to replace traditional batteries as the power source for WSNs in IoT. The various applications of these generators are expected to increase, and considering the demand, these devices may become commercially successful. Thermoelectric power generation has not yet reached wide-scale application; however, this situation may be changed by improving energy harvesting devices.
